# A Chromosome-Scale Assembly of the *Bactrocera cucurbitae* Genome Provides Insight to the Genetic Basis of *white pupae*

**DOI:** 10.1534/g3.117.040170

**Published:** 2017-04-20

**Authors:** Sheina B. Sim, Scott M. Geib

**Affiliations:** United States Department of Agriculture, Agricultural Research Service Daniel K. Inouye United States Pacific Basin Agricultural Research Center, Tropical Crop and Commodity Research Unit, Hilo, Hawaii 96720

**Keywords:** Tephritid fruit flies, Genetic sexing, Sterile Insect Technique, Mendelian genetics, genomics, whole genome sequencing, chromosome assembly, QTL, linkage mapping, synteny, Diptera, *Drosophila*, genotyping

## Abstract

Genetic sexing strains (GSS) used in sterile insect technique (SIT) programs are textbook examples of how classical Mendelian genetics can be directly implemented in the management of agricultural insect pests. Although the foundation of traditionally developed GSS are single locus, autosomal recessive traits, their genetic basis are largely unknown. With the advent of modern genomic techniques, the genetic basis of sexing traits in GSS can now be further investigated. This study is the first of its kind to integrate traditional genetic techniques with emerging genomics to characterize a GSS using the tephritid fruit fly pest *Bactrocera cucurbitae* as a model. These techniques include whole-genome sequencing, the development of a mapping population and linkage map, and quantitative trait analysis. The experiment designed to map the genetic sexing trait in *B. cucurbitae*, *white pupae* (*wp*), also enabled the generation of a chromosome-scale genome assembly by integrating the linkage map with the assembly. Quantitative trait loci analysis revealed SNP loci near position 42 MB on chromosome 3 to be tightly linked to *wp*. Gene annotation and synteny analysis show a near perfect relationship between chromosomes in *B. cucurbitae* and Muller elements A–E in *Drosophila melanogaster*. This chromosome-scale genome assembly is complete, has high contiguity, was generated using a minimal input DNA, and will be used to further characterize the genetic mechanisms underlying *wp*. Knowledge of the genetic basis of genetic sexing traits can be used to improve SIT in this species and expand it to other economically important Diptera.

The melon fly, *Bactrocera cucurbitae* (Coquillett; order Diptera: family Tephritidae) (Figure 1A), is a destructive agricultural pest known to infest >100 varieties of fruit and vegetable crops (Dhillon *et al.* 2005; Doharey 1983; White and Elson-Harris 1992). It has been defined as a category A pest due to its destructiveness as a specialist oligophage that has become established outside of its native range (Vargas *et al.* 2015). Its current geographic range includes its native range in the Indian subcontinent and introduced range in Asia, Australia-Oceania, Hawaii, South America, and Africa (Dhillon *et al.* 2005; Bezzi 1913; Wu *et al.* 2011). To protect the range of *B. cucurbitae* hosts in the mainland United States, an industry that generates $4.5 billion yearly in California alone (Ross 2015), *B. cucurbitae* is the target of strict and costly exclusion, detection, quarantine, and eradication protocols that are enforced to prevent its establishment.

**Figure 1:**
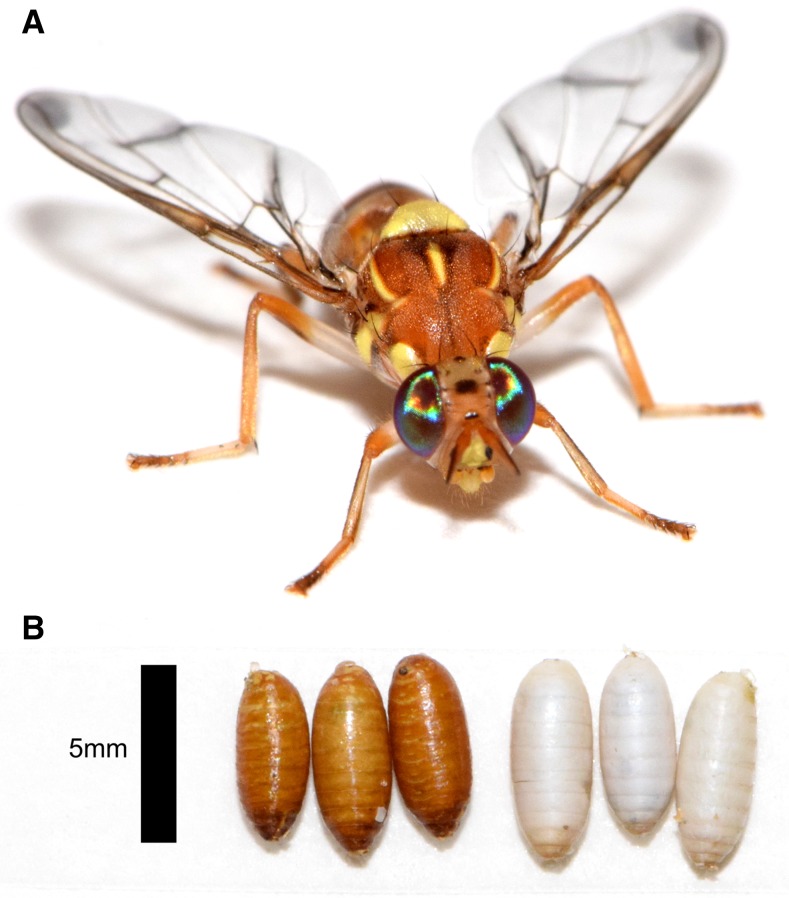
*B. cucurbitae*. (A) Front view of an adult female *B. cucurbitae*. (B) A male heterozygous at the *wp* locus featuring a wild-type brown pupal case (left) and a female homozygous for the *wp* allele featuring a white pupal case (right). Pupal color phenotype is the sexing trait used to sort males from females in the T1 Melon genetic sexing strain.

A current management strategy used for exclusion and eradication, as a component of an area-wide integrated pest management program for tephritid fruit flies, is the sterile insect technique (SIT). The release of insects for SIT has been effective in the control of several pest species, including tephritid fruit flies such as the Mediterranean fruit fly *Ceratitis capitata*, Mexican fruit fly *Anastrepha ludens*, and *B. cucurbitae*. Due to its efficacy and reduced nontarget effects, there is increased interest in expanding the use of this technique to several important tephritid genera within the United States and globally (Knipling 1955). SIT facilitated eradication of *B. cucurbitae* from the island of Rota (Steiner 1965; Steiner *et al.* 1965) and the southwestern islands of Japan (Koyama *et al.* 2004) using a sterilized bisexual strain in which melon flies were mass-reared, sterilized, and both males and females were released. While these previous eradication efforts were successful, the release of both sexes for SIT is not preferred due to released females competing with the wild females to mate with sterile males, the loss of commodity crops due to oviposition damage by sterile females, and the increased cost of rearing both males and females. All of these factors contribute to lower the potential efficiency of bisexual SIT treatment (Rendon *et al.* 2004; Rossler 1979; Whitten 1969; Zepeda-Cisneros *et al.* 2014).

The ability to mass-rear target species for male only releases is critical to cost-effectiveness and efficacy of SIT programs, and is facilitated by the availability of a genetic sexing strain (GSS). A GSS is a strain in which individuals can be separated by sex prior to adulthood using a sex-linked phenotype (Franz 2005). Classical GSS requires a marker that can be selected upon for separation by sex and a Y-autosome translocation to make the trait sex-linked (Franz 2005). In the melon fly GSS (T1 Melon strain), pupal color is sexually dimorphic, with females having an atypical white pupal case and males having a wild-type brown pupal case (Figure 1B) (McInnis *et al.* 2004). This pupal color variant was first described by McCombs *et al.* (1996) and is similar in phenotype to the previously identified white pupae trait in the oriental fruit fly *B. dorsalis* (McCombs and Saul 1995) and the Mediterranean fruit fly *C. capitata* (Rossler 1979; Rossler and Rosenthal 1992; Franz *et al.* 1994). *White pupae* (*wp*) is an autosomal recessive trait, and thus requires individuals to be homozygous for the mutation to exhibit the mutant phenotype. Although autosomal, *wp* has been made sex-linked by an irradiation-induced translocation that occurred between the tip of the autosome containing the *wp* gene and the Y chromosome in a male that was propagated in a stable *wp* line (McInnis *et al.* 2004). The translocation harbors a wild-type *wp* allele and maintains heterozygosity and a wild-type pupal color phenotype (Figure 2A). By contrast, females in the GSS line do not carry the translocation and are homozygous for the mutation. In wild-type flies, individuals are homozygous for the wild-type alleles and lack the known chromosomal rearrangement (Figure 2B). While GSS strains can also be developed based on transgenic genomic modifications (Ant *et al.* 2012; Schetelig and Handler 2012a,b), the adoption of these systems is not widespread due to negative public perception (Einsiedel 2005) and regulatory considerations associated with releasing transgenic organisms. Thus, there is still relevance in exploring classical GSS systems in new species.

**Figure 2:**
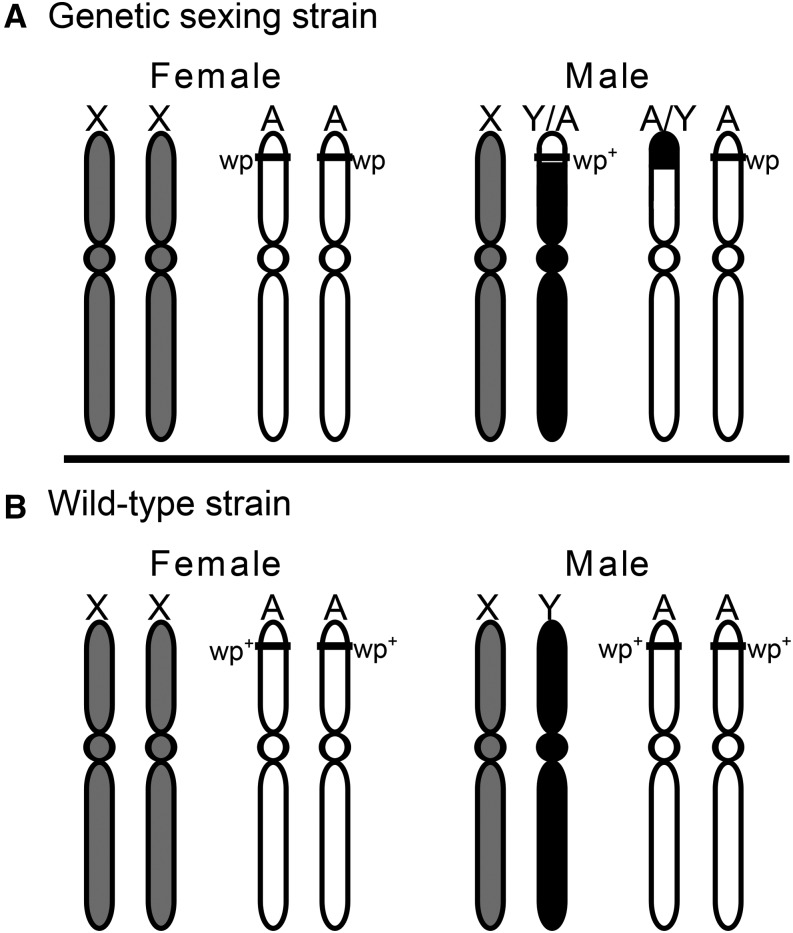
Sex chromosomes and autosome of genetic sexing strain and wild-type *B. cucurbitae*. (A) Drawing of the sex chromosomes and the autosome containing the *wp* gene for female and male T1 Melon *B. cucurbitae*. The location of the *wp* gene has been crudely mapped to the tip of one of the autosomes, and a translocation between the Y-chromosome and the autosome harbors a wild-type allele (*wp*^+^) in males and are heterozygous at the locus in contrast to females, who are homozygous for the *wp* mutation. (B) Drawing of the sex chromosomes and the autosome containing the gene for female and male wild-type *B. cucurbitae*; wild-type individuals lack the *wp* mutation. Figure adapted from Franz (2005).

Although the T1 Melon GSS has existed for over a decade, few foundational genomic tools exist for *B. cucurbitae*, and the genetic basis for *wp* is unknown. The purpose of this study was to use an integrative assembly and mapping approach that merges classical genetics with modern genomic techniques to characterize the genetic basis of *wp*. This was accomplished by generating, in parallel, a high-quality draft whole-genome assembly and linkage map for *B. cucurbitae*. The process of scaffolding for assembly is then iterated through the use of the *B. cucurbitae* linkage map to further assemble the genome assembly into a chromosome-scale assembly. The usage of linkage mapping for *de novo* genome assembly has been previously described as a viable approach (Tang *et al.* 2015; Paina *et al.* 2016). A variation of this technique has been used to improve the existing *Heliconius melpomene* genome assembly (Davey *et al.* 2016) and has been previously used to assemble the genome of a nonmodel organism, *Plutella xylostella*, through usage of comparative genomics with *Bombyx mori* (Baxter *et al.* 2011).

The resulting assembly is of high quality according to various quality metrics such as contiguity and completeness of gene set, and shows a high level of synteny with chromosome arms of *Drosophila melanogaster*. All of these products lay the foundation for tools to expand classical genetic sexing systems in pest dipterans.

## Materials and Methods

### Fly samples

#### Laboratory colonies and rearing conditions:

Specimens used for whole-genome sequencing and assembly were derived from the T1 *wp* translocated line of *B. cucurbitae*. Adult flies were reared under similar conditions as described in Vargas (1989), with minor amendments. In this study, adult *B. cucurbitae* were reared in a 25-cm cubical cage in a room maintained at 25° with 65% humidity, and maintained on a diet of a 3:1 ratio of white sugar and yeast hydrolysate. Melon fly larvae were reared in the same environmental conditions as adults and given a diet consisting of 30% wheat mill feed, 7% granulated white sugar, 3.5% torula yeast, and 59.5% water with negligible amounts of the preservatives nipagen and sodium benzoate.

#### DNA extraction methods:

DNA extraction of adult *B. cucurbitae* for sequencing was performed using previously published methods (Sim *et al.* 2016). Briefly, whole adult fly samples were homogenized in tissue lysis buffer using a FastPrep 24 homogenizer (MP Biomedical, Santa Ana, CA) for 20 sec at 4.0 m/sec. Homogenized samples were incubated in a 55° water bath for 3 hr, followed by DNA extraction on a Kingfisher Flex 96 automated extraction instrument (Thermo Scientific, Waltham, MA), using standard protocols and a Mag-Bind Tissue DNA KF Kit (Omega Bio-Tek, Norcross, GA). The quantity and quality of the extracted DNA sample was determined using the High Sensitivity Genomic DNA Analysis Kit on a Fragment Analyzer (Advanced Analytical, Ankeny, IA).

### Whole-genome sequencing

#### Library construction, sequencing, and assembly:

Library preparation methods were used to optimize genome assembly with the ALLPATHS-LG assembler. A 180-bp insert Illumina TruSeq fragment library was constructed from 500 ng DNA extracted from a single GSS male. This individual was the F2 offspring of an isolated mating between two GSS parents. Additionally, two Illumina Nextera mate-pair libraries targeting a 3- and 8-kb insert size, respectively, were constructed using DNA from a pool of sibling GSS males that were derived from the same isolated mating parents as the individual in the fragment library. The fragment and mate-paired libraries were sequenced using 2 × 100 bp sequencing on the Illumina HiSeq 2500 in High Output mode. The SRA accessions for each library, along with additional read counts and approximate read depths, are presented in Table 1. Raw reads from the fragment and mate pair libraries were used to construct a scaffold assembly using ALLPATHS-LG (v.44837) (Gnerre *et al.* 2011; Ribeiro *et al.* 2012) with default parameters, with the exception of addition of “HAPLOIDIFY = TRUE.” Kmer-based error correction of the fragment library was performed prior to assembly as part of the ALLPATHS-LG pipeline. The draft scaffold assembly was integrated with linkage data (described in more detail in *Linkage mapping and QTL analysis*) and placed into chromosome-scale superscaffolds.

**Table 1:** Raw and used reads per Illumina HiSequation library type

BioSample	Library Type	Raw Reads, M	Base Pairs, Gb	Coverage
SAMN03010452	180 bp Fragment	212.7	21.3	66.2×
SAMN03010453	3 kb Jumping	169.9	17	52.9×
SAMN03010454	8 kb Jumping	63.8	6.4	19.9×

#### Genome annotation and orthology analysis:

Structural and functional annotation of genes was performed with the NCBI Eukaryotic Genome Annotation Pipeline. This automated annotation pipeline utilized transcript evidence from existing RNA-seq data for *B. cucurbitae* (Sim *et al.* 2015), as well as RNA-seq data from several other *Bactrocera* species. In addition, NCBI RefSeq protein sets for *C. capitata*, *D. melanogaster*, and *Musca domestica*, and 78,566 NCBI GenBank Insecta proteins were aligned to the genome and used to inform gene model prediction using the NCBI eukaryotic gene prediction tool *GNOMON*. Prior to this study, there were no RefSeq proteins for this species curated in NCBI. An overview of the annotation release (*B. cucurbitae* annotation release 100) is available online at http://www.ncbi.nlm.nih.gov/genome/annotation_euk/Bactrocera_cucurbitae/100/.

The completeness of the genome and gene set was analyzed by identifying the number of arthropod Benchmark Universal Single-Copy Orthologs (BUSCOs) (Simao *et al.* 2015), a set of 2675 proteins considered to be conserved in nearly all arthropods. Using BUSCO v1.1b1, the predetermined arthropod BUSCO database was used with the *B. cucurbitae* scaffolded genome assembly, as well as the NCBI RefSeq annotation gene set. The proportion of complete BUSCOs found in the genome assembly (“-m all” option) and in the gene set (“-m OGS” option) were compared with other notable arthropod genomes and gene sets previously reported (Simao *et al.* 2015). A comparison of orthologous genes between *B. cucurbitae* and 17 arthropod species was performed and described in the Supplemental Material, File S1 with the sources for the genomes and gene sets listed in Table S1.

### Double-digest RAD library preparation, sequencing, and single nucleotide polymorphism analysis

#### Crossing scheme:

Two inbred lines, the T1 white pupae strain and a wild-type laboratory colony, were used to generate the five mapping populations represented in the double-digest RAD (ddRAD) library. First, T1 Melon white pupae females were isolated from the T1 Melon wild-type brown pupae males in the pupal stage. Wild-type colony males were separated from wild-type colony females shortly after eclosion. At 21 d posteclosion, at a point when melon fly adults were sexually mature, a single T1 Melon female and a single wild-type colony male were paired in 250-ml mating cups and provided with food and water. Eggs from females were collected by placing an oviposition cup containing a sponge soaked in sieved tomato juice to stimulate egging. Eggs were collected from females until they stopped producing eggs, and live adult mating pairs were snap frozen in liquid nitrogen and stored in 99% ethanol until nucleic acid extraction. If either parent died during egg production, that cross and resulting offspring were discarded to avoid having to use poor quality DNA derived from dead insects. Eggs collected from isolated mating pairs were placed on larval diet in 1-oz cups. Larvae were allowed to develop, and prior to pupation, melon flies exhibit a jumping or “popping” behavior where they exit their larval media. Larvae “popped” into a provided sand substrate where they pupated. Pupae were collected from the sand substrate, pupal color phenotype was recorded, and newly eclosed adults were sexed. Phenotyped virgin adults were maintained in isolation for use in subsequent generations. These crosses were performed in excess, discarding low performing mating pairs (*i.e.*, those not producing eggs or where individuals died), and only the most productive mating pairs were maintained for subsequent generations.

**Figure 3:**
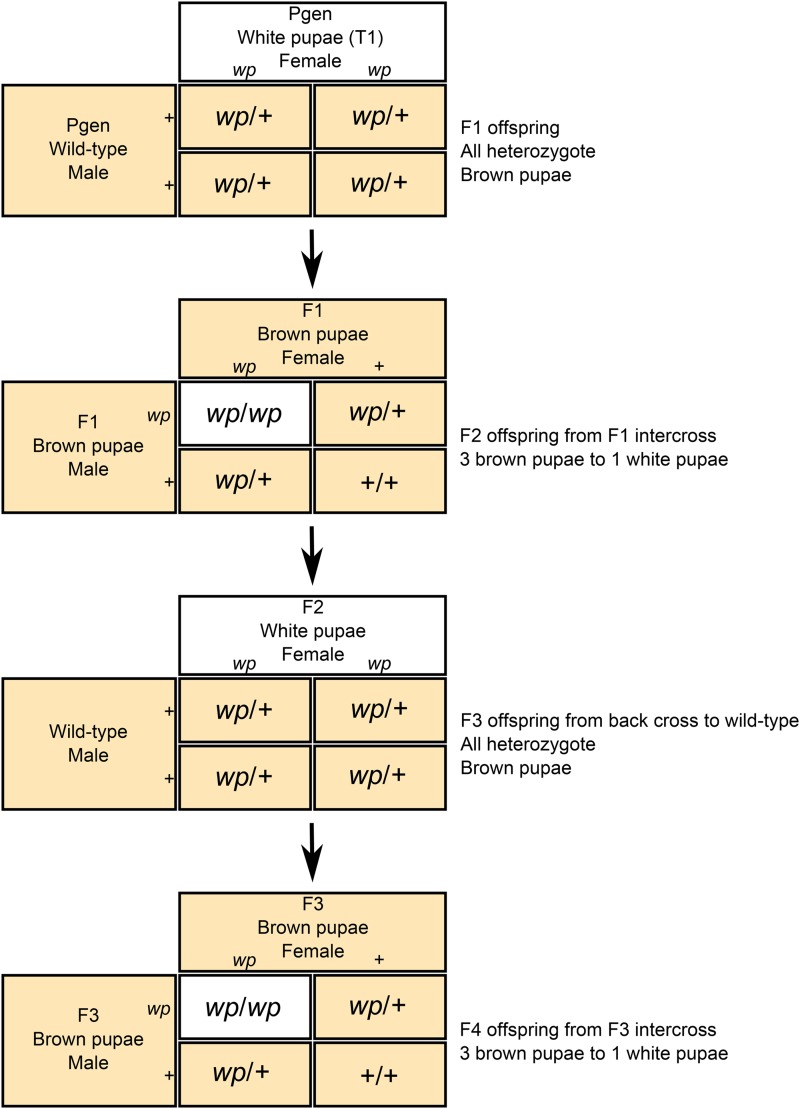
Crossing scheme used to generate F4 mapping population. Virgin adult females from the *B. cucurbitae* white pupae genetic sexing strain were mated in isolation with males from the wild-type laboratory colony. The white pupae trait is autosomal recessive; resulting F1 progeny will all have a wild-type brown pupal color phenotype. In F2 progeny from isolated intercrossing between F1 full sibs, the pupal color phenotype will segregate at a 3:1 ratio of wild-type brown pupae to white pupae. White pupae F2 females were backcrossed to wild-type laboratory colony males. This increases the proportion of the wild-type alleles genome in subsequent offspring. Like the F1 progeny, the F3 progeny will all have a wild-type brown pupal color phenotype and full sibs will be intercrossed to produce an F4 mapping population comprised of female and male wild-type brown pupae and white pupae individuals.

The crossing scheme employed (Figure 3) produced recombinant offspring with nonsex-linked variation in pupal color (white or wild type) in an inbred wild-type background. To generate the mapping population, virgin Parental (P) generation T1 white pupae females were mated with virgin wild-type colony males in isolated crosses. The resulting F1 population had a wild-type brown pupae phenotype and were heterozygous at the white pupae locus. These F1 individuals were intercrossed to recover the white pupae phenotype in the F2 generation. The resulting white pupae F2 females were backcrossed to wild-type colony males in isolated matings. The resulting F3s, which were heterozygous at the *wp* locus, were intercrossed to recover the white pupae phenotype. This method of generating a mapping population decoupled the white pupae phenotype from sex due to the exclusion of the reciprocal translocated autosome and Y chromosome through usage of wild-type colony males, which have a wild-type chromosomal arrangement. This crossing scheme was replicated and produced many sibships. The five most productive sibships, including parents and all offspring, were used to generate ddRAD libraries for calculating a linkage map and performing trait mapping.

#### ddRAD library preparation:

Sibships comprised of individuals segregating for the white pupae trait along with their F3 parents, F2 grandparents, and male and female flies from both the T1 and wild-type laboratory colonies were used to construct a double-digest restriction site–associated DNA library, using methods described by Peterson *et al.* (2012). Briefly, ∼250 ng DNA per individual was digested using the restriction enzymes *Nla*III and *Mlu*CI. One of 48 unique barcoded adapters were ligated to the restriction overhang, generating inline barcodes, and subpools of samples containing these 48 barcodes were generated and size-selected using a 1.5% agarose gel cassette on a Blue Pippin electrophoresis unit (Sage Science, Beverly, MA), with a target size selection of “narrow 400 bp.” The final PCR amplification step was run for 10 cycles, during which a second barcode was added in the Illumina i7 location for each subpool, and PCR products were cleaned using solid-phase reversible immobilization beads at a 1.5:1 ratio of PEG containing bead solution to sample volume (DeAngelis *et al.* 1995; Rohland and Reich 2012). The final sublibraries were analyzed for quantity and size distribution using the NGS Fragment Analysis Kit on a Fragment Analyzer (Advanced Analytical) and pooled at equal molar ratios to generate the final library. Four libraries were created in this manner, including a total of 166 individual samples. The ddRAD libraries were each subjected to 100-bp single-end sequencing on a lane of an Illumina HiSequation 2500 Sequencer run in Rapid-Run mode.

#### Single nucleotide polymorphism identification and genotyping:

Single nucleotide polymorphisms (SNPs) were identified and genotypes were called for each individual using the Stacks version 1.35 pipeline (Catchen *et al.* 2011, 2013). Briefly, the Stacks *process_radtags.pl* script was used to demultiplex the inline barcodes for each of the four i7 Illumina indexed outputs. This resulted in one fastq formatted file per individual which was then mapped to the *B. cucurbitae* draft scaffold assembly using the BWA-mem algorithm described by Li (2012) which produced one SAM formatted file per individual. The Stacks *ref_map.pl* script was used to generate a catalog of sequence loci of known genomic position (based on reference assembly), and a catalog of polymorphisms based on sequence variation. Finally, the stacks *populations.pl* script was used to identify the genotype of every individual at every locus in the catalog requiring a minimum read depth of 10 reads per locus. Loci for which there were >75% missing genotypes were filtered out of the data set. Weir and Cocheram Fst values were calculated between the wild-type colony and the T1 Melon colony for each SNP locus identified in the superscaffold assembly using the “–fst” function of Plink v1.9 (Chang *et al.* 2015), and a resulting Manhattan plot was generated using the qqman package in R (Turner 2014; R Core Team 2015). The average heterozygosity for both colonies was estimated also using the “–het” function of Plink v1.9, and box plots with 95% mean and confidence intervals were generated using the “plot” function of R (R Core Team 2015).

### Linkage map construction, QTL analysis, and superscaffolding

#### Linkage mapping and QTL analysis:

The SNP genotypes obtained from the Stacks pipeline were used to estimate a linkage map featuring chromosome-scale linkage groups. This analysis was performed using the program Lep-MAP2 (Rastas *et al.* 2013, 2015) which was written specifically for use with genome-wide SNP data and organisms with achiasmatic meiosis, such as insects in the orders Lepidoptera and Diptera. Using Lep-MAP2, genome-wide SNP genotypes from all parents and progeny from the five mapping populations were filtered for Mendelian errors using custom scripts and the *Filtering* module. Loci were then assigned to linkage groups using the *SeparateChromosomes* module. A minimum of 50 loci was required for a linkage group to be reported. Ungrouped SNP loci were included using the *JoinSingles* module, and the *OrderMarkers* module was used to order the markers within each linkage group defined by *SeparateChromosomes*. Due to the lowered and nearly absent recombination in male Diptera (Morgan 1914; Rossler 1982), the initial recombination rate for males was set to 1e−9 in the *OrderMarkers* module, and the female recombination rate was set to 0.05. SNP loci linked to the *wp* locus were identified using R-QTL, implementing a binary QTL model. A permutation test using 10,000 permutations was performed to identify significant loci above the threshold of 1e−4 (Broman 2003; Broman *et al.* 2003; Xu and Atchley 1996). The SNP loci displaying the strongest linkage to the white pupae phenotype, as determined by a permutation test, was used to design TaqMan SNP genotyping assays to differentiate between white pupae and wild-type brown pupae individuals (File S2).

#### Superscaffolding and analysis for synteny:

Superscaffolding of the ALLPATHS-LG scaffold assembly was performed using ALLMAPS, which integrated the information from the linkage map to anchor, order, and orient scaffolds into a new assembly (Tang *et al.* 2015). The *merge* function from ALLMAPS was used to generate a BED formatted file from linkage information. This BED file was then analyzed using the *path* function from ALLMAPS, which sorted scaffolds based on their average position in the linkage map. Accuracy of the map is evaluated by calculating the ratio between the longest monotonic subsequence and the total number of markers (ρ). This ratio will range from 0 to 1, with a ρ = 1 signifying perfect collinearity between the assembled scaffold order and the linkage map. The output of ALLMAPS includes a superscaffolded fasta formatted file, plots visualizing the assembly of each chromosome, and a chain formatted file which was used to lift over the positions of the annotations in the scaffold assembly to the new superscaffolded assembly, using the *liftover* command line utility associated with the UCSC genome browser (Kent *et al.* 2002).

A comparison of the physical location of single-copy orthologs between the *B. cucurbitae* superscaffold assembly and *D. melanogaster* (FlyBase r6.07) was illustrated using the R package RCircos (Zhang *et al.* 2013), and analysis for collinearity was performed by MCScanX (Wang *et al.* 2012). The required input homology file for RCircos and MCScanX was constructed from the single-copy orthologs identified between *B. cucurbitae* and *D. melanogaster* from the output of the OrthoMCL (Li *et al.* 2003) analysis. MCScanX was used to generate a collinearity file containing pairwise collinear blocks between the two genomes, and RCircos was used to draw a connecting line between orthologous genes in a circular plot between the *B. cucurbitae* and *D. melanogaster* chromosomes. Following the precedent set by Painter (1934) in *Drosophila* and previous polytene chromosome mapping studies in *B. cucurbitae* (Shahjahan and Yesmin 2002; Zacharopoulou and Franz 2013), chromosomes were named 2–6 in descending order from largest (in megabases) to smallest. While previous cytogenetic maps exist for this species, they cannot be integrated with our genetic linkage map because they did not physically map markers onto each chromosome to serve as anchor points, so chromosome numbering may not be conserved between the two mapping methods (Shahjahan and Yesmin 2002; Zacharopoulou and Franz 2013).

### Data availability

All raw sequences associated with the contig and scaffold assemblies and the most current gene set are stored and curated at NCBI under BioProject accession number PRJNA314357. Additionally, these data are also hosted in the USDA National Agriculture Library I5 K Workspace (https://i5k.nal.usda.gov/). Demultiplexed ddRAD sequences are also stored and curated at NCBI under BioProject accession number PRJNA314357. Supporting data, including the chromosome-scale assembly, rQTL analysis file, raw linkage map, .vcf, lifted .gff3, and .chain file used to lift the coordinates of the scaffold .gff3 to the chromosome-scale .gff3, are available at the USDA Ag Data commons (https://data.nal.usda.gov) under DOI 10.15482/USDA.ADC/1329913. Detailed descriptions of all supplemental files including references used in supplemental files is presented in File S3.

## Results and Discussion

### Whole-genome sequencing and assembly

To investigate the white pupae trait in the T1 GSS colony of *B. cucurbitae*, first a high-quality draft genome assembly was created, utilizing ALLPATHS-LG. To accomplish this, the genome of one adult *B. cucurbitae* male was sequenced to 66.2× fragment library coverage, with additional mate-paired libraries made from a pool of its full sibling brothers used for scaffolding (Table 1). All libraries were subjected to 2 × 100-bp sequencing and run on a single lane of an Illumina HiSequation 2500 in High Output mode. The initial scaffold assembly generated by ALLPATHS-LG had an N50 of 1.4 Mb, a total of 5572 scaffolds, and a total assembly length of ∼364 Mb (Table 2). This genome assembly length was very close to the estimated genome size of 373 Mb (an estimate based on kmer abundance), which demonstrates the completeness of the assembly. Gene annotation statistics obtained as a result of the NCBI Eukaryotic Genome Annotation Pipeline are shown in (Table 3).

**Table 2:** Assembly summary statistics

Assembly Type	Count	N50	Total length, Mb
Contig	43,002	43 Kb	316.3
Scaffold	5572	1.4 Mb	363.6 (with gaps)
Chromosome	5	34.1 Mb	244.9 (with gaps)

**Table 3:** Gene annotation summary statistics

Feature	Count	Mean Length, bp	Median Length, bp	Min. Length, bp	Max. Length, bp
Genes	13,286	13,572	2998	71	692,581
All Transcripts	22,048	2653	1983	43	60,413
mRNA	20,741	2763	2067	207	60,413
Misc. RNA	220	2996	2445	122	13,488
tRNA	407	74	73	71	84
lncRNA	680	745	538	43	6750
Single-Exon Transcripts	1062	1366	1118	246	11,133
CDSs	20,741	2093	1476	120	59,988
Exons	77,461	423	234	2	19,461
Introns	62,246	3014	109	30	583,620

**Figure 4:**
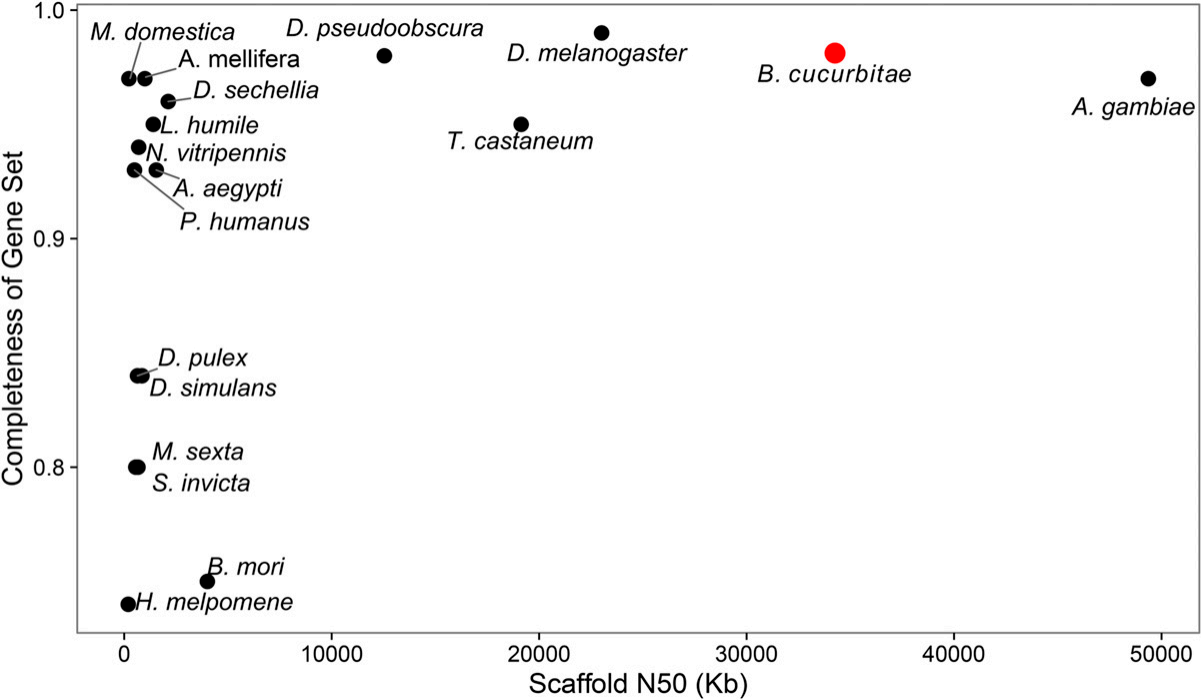
Comparison of continuity in terms of N50 and proportion of complete BUSCOs in the gene set. *B. cucurbitae* (red circle) ranks high in continuity (*x*-axis) and proportion of complete BUSCOs (*y*-axis) compared to notable arthropods.

Analysis of completeness using BUSCO showed the assembly contained 98% of known arthropod BUSCOs, and compared to the genomes of other notable arthropods, is among the most complete in terms of presence of conserved orthologs (Table 4). An analysis of assembly continuity (superscaffold N50) against completeness of the gene set in terms of the proportion of found complete BUSCOs show *B. cucurbitae* among the most complete, and high continuity among notable arthropod genomes and gene sets (Figure 4). BUSCO-derived single-copy orthologs were used to place the genomes within a phylogenetic relationship. A summary of the number of orthologous groups shared between *B. cucurbitae* and decreasingly specific taxonomic ranks is summarized in Figure S4. The data set is available under NCBI Assembly Accession GCF_000806345.1, BioProject PRJNA259565, and RefSeq accession PRJNA273817. The samples used in the whole-genome assembly are identified as BioSample SAMN03083541 at NCBI. In addition, this genome is hosted in the USDA National Agriculture Library I5 K Workspace (https://i5k.nal.usda.gov/), which provides a full genome browser, BLAST suite, domain searching, and other tools.

**Table 4:** N50 scaffold lengths and BUSCO completeness of genome and gene set for selected arthropod species

Species	Scaffold N50, Kb	Completeness, Genome	Completeness, Gene Set
*Drosophila melanogaster*	23,011	0.98	0.99
*Bactrocera cucurbitae*	34,136	0.92	0.98
*Drosophila pseudoobscura*	12,541	0.96	0.98
*Anopheles gambiae*	49,364	0.93	0.97
*Apis mellifera*	997	0.93	0.97
*Musca domestica*	226	0.91	0.97
*Drosophila sechellia*	2123	0.96	0.96
*Linepithema humile*	1402	0.92	0.95
*Tribolium castaneum*	19,135	0.95	0.95
*Nasonia vitripennis*	698	0.76	0.94
*Aedes aegypti*	1547	0.86	0.93
*Pediculus humanus*	497	0.92	0.93
*Daphnia pulex*	642	0.83	0.84
*Drosophila simulans*	857	0.85	0.84
*Manduca sexta*	664	0.81	0.80
*Solenopsis invicta*	558	0.74	0.80
*Bombyx mori*	4008	0.73	0.75
*Heliconius melpomene*	194	0.77	0.74

The draft *B. cucurbitae* whole-genome assembly and annotated gene set was generated using a very modest budget, yet is as high in contiguity and level of completeness as other genomes of similar size and complexity. Various quality metrics, such as scaffold N50 size, number of scaffolds, and an assembled genome size similar to the estimated genome size based on kmer abundance, provide evidence that the experimental approach for generating the assembly was successful. One of the hurdles for genome assembly in *B. cucurbitae* is the limited amount of genomic DNA that can be extracted from individual organisms, and this study demonstrates what can be accomplished using minimal quantities of input DNA that can be obtained from organisms similar in size to *B. cucurbitae* and other Tephritidae.

### Mapping population and SNP genotyping

To generate a linkage map and to identify the genomic position of the *wp* locus in *B. cucurbitae*, an F4 mapping population was generated using an isolated-mating crossing scheme (Figure 3). The crossing of individual *wp* females from the T1 GSS colony with individual males from a wild-type colony resulted in the segregation of white pupae alleles and placed the mutant white pupae and wild-type brown pupae phenotypes in a common genetic background. Five sibships were generated using this isolated-mating crossing scheme, and these sibships were comprised of white pupae and wild-type individuals. The proportion of wild-type brown pupae individuals to white pupae individuals was recorded at every generation. The P generation was comprised of wild-type brown pupae males and white pupae females. As expected, the F1 and F3 generation had 100% brown pupae phenotype. The F2 and F4 generation that were the progeny of F1 and F3 full sibling intercrosses, respectively, was comprised of brown and white pupae individuals at a ratio that did not significantly deviate from 3:1 (adjusted *P*-value < 0.05) (Table 5), which is the ratio expected from single heterozygote crosses. In each generation, there was no significant bias in sex ratio. All sibships were derived from full sibling matings and all full sibling pairs were derived from one isolated mating pair.

**Table 5:** Ratios of wild-type to white pupae individuals in F2 and F4 sibships

Cross Number	Generation	Total	Brown	White	$\chi^{2}$	*P*-Value
18	F2	63	55	8	1.27	0.26
18.6.6	F4	34	32	2	1.66	0.20
18.6.8	F4	52	42	10	0.23	0.63
18.6.16	F4	57	45	12	0.14	0.70
18.6.44	F4	45	28	17	0.98	0.32
18.6.58	F4	41	32	9	0.05	0.82

To rapidly genotype individuals from the mapping population, ddRAD libraries were constructed and sequenced. Demultiplexed ddRAD sequences are stored and curated on NCBI under BioProject PRJNA314357, BioSample SAMN04543723, and SRA study accession number SRP071607. The number of raw reads, reads removed due to lack of restriction site, reads removed due to low quality, and number of reads retained are included in Table S2. A total of 25,300 SNPs were identified from 7143 unique RAD sites on 948 unique scaffolds. Genome-wide Fst values show 45 SNP loci with fixed differences (Fst = 1.0) between the wild-type colony and the T1 Melon colony across the genome (Figure S1). An estimate of the level of inbreeding between the wild-type colony and the T1 Melon colony shows on average no inbreeding (F coefficient < 0) for all SNPs identified in both colonies (Figure S2).

The number of fixed differences between the two colonies in addition to the high Fst values across the genome indicate a high level of divergence between the T1 Melon colony and the wild-type colony. The high genetic variation between the two colonies suggests that a genome-wide association study would not have necessarily identified the trait of interest, (*wp*) if only colony flies were genotyped and no isolated crosses and backcrosses were performed. The estimation of mean heterozygosity does not indicate genome-wide levels of inbreeding and shows that genetic variation still exists in these colonies despite their limited population size and lack of immigration. However, it is important to note that the amount of inbreeding may be underestimated in this study due to the fact that only variable loci are used in this analysis and no wild flies were genotyped for comparison.

### Linkage map construction and QTL mapping

**Figure 5:**
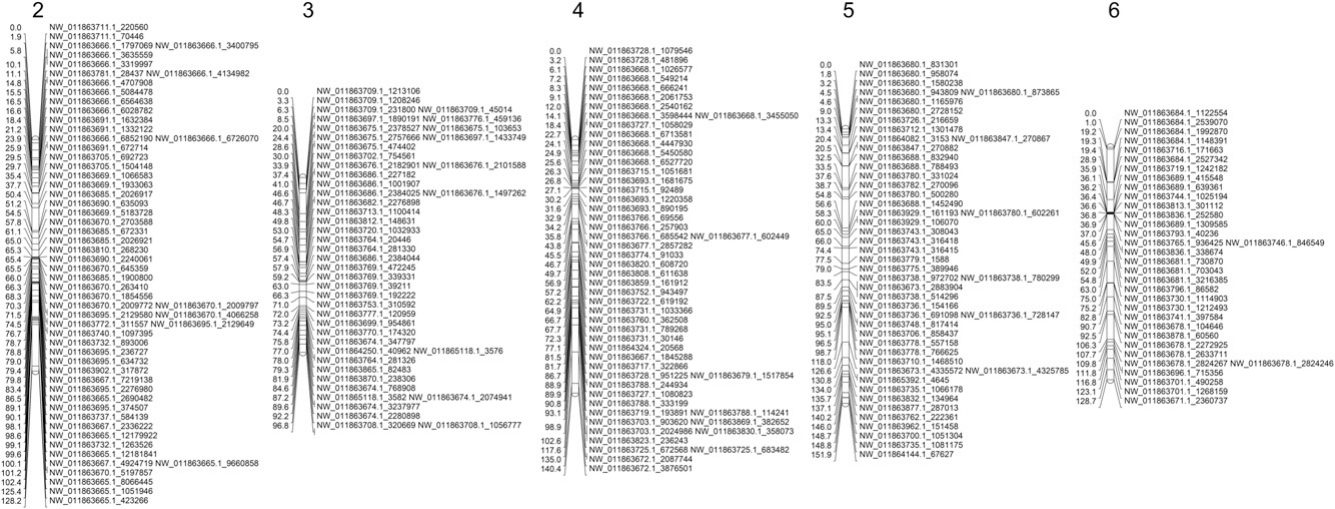
Linkage map. Genetic map depicting size of linkage group in centimorgan and distribution of SNPs in five linkage groups.

Linkage analysis of the five mapping populations placed 944 SNPs from 169 unique scaffolds onto five linkage groups ranging from 39 to 60 Mb in size and represent 65.7% of the assembled genome. Identified linkage groups ranging from 97 to 140 cM in size, their sizes, and distribution of SNP loci are illustrated in Figure 5. Linkage groups were numbered in sequential order from largest (in terms of size in base pairs) to smallest.

**Figure 6:**
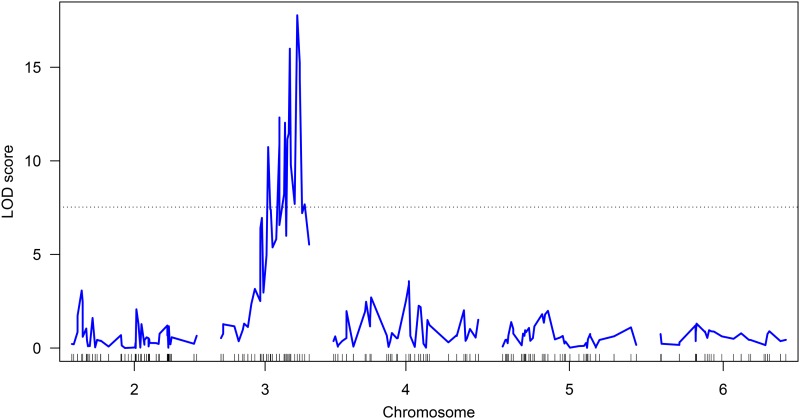
QTL map. QTL analysis using the binary interval mapping model. Results indicate that the pupal color phenotype is tightly linked to loci on the autosome chromosome 3. A permutation test performed with 100,000 permutations identified loci showing significant linkage to the phenotype (*P* < 1e−4).

Analysis of linkage between SNP loci mapped to linkage groups and pupal color phenotype using a binary QTL model and permutation test showed significant linkage (p < 1e−4) between loci on scaffolds NW_011863770.1 and NW_011863674.1, which are ∼0.900 and 3.9 Mb in length, respectively. The most significant tightly linked locus was on scaffold NW_011863674.1 at position 768,903, with an LOD score of 17.78 (Figure 6). Though tightly linked to the white pupae trait, the SNP loci with the highest LOD scores are unlikely to be the actual causative mutation that confers the white pupae phenotype, however the *wp* gene and mutation are likely located near those SNPs in the genome. The discrimination assay described in File S2 was validated on colony white pupae and brown pupae individuals, and showed consistency in being able to discriminate between the homozygote white pupae females from the T1 Melon GSS colony, the wild-type brown pupae males from the T1 Melon GSS colony, and the males and females from the wild-type colony (Figure S5). This result demonstrates a high degree of linkage between the SNP loci identified through QTL analysis and *wp*, and the genes in close physical proximity to the linked loci can be further interrogated in future studies to identify the *wp* gene.

There are a total of 797 genes located within the genomic region containing significant SNP loci. Of these 797 genes, 11 have function related to cuticular structure or sclerotization (Table S3), but none are in the arthropod melanization pathway (Fukushi 1967; Sugumaran *et al.* 1992; De Gregorio *et al.* 2002; True 2003; Cerenius and Soderhall 2004; van’t Hof and Saccheri 2010; Du *et al.* 2017). Interrogation of the genome yielded orthologs for all the known genes in the arthropod melanization pathway, such as genes producing prophenoloxidase, prophenoloxidase-activating proteinase, NBAD hydrolase, NBAD synthase, laccase, yellow, serine protease inhibitor, *etc*. However, none of these genes are found in the significant QTL region or are found on unplaced scaffolds. It is important to note that although most of the genes in the significant QTL region do not have obvious functions related to melanization, cuticle structure, or cuticle sclerotization, they cannot be excluded as candidates as there are likely many additional genes that have functions up- or downstream of the melanization pathway that may be involved specifically in melanization of the pupal sheath or case in Tephritidae.

### Superscaffolding and synteny analysis

**Figure 7:**
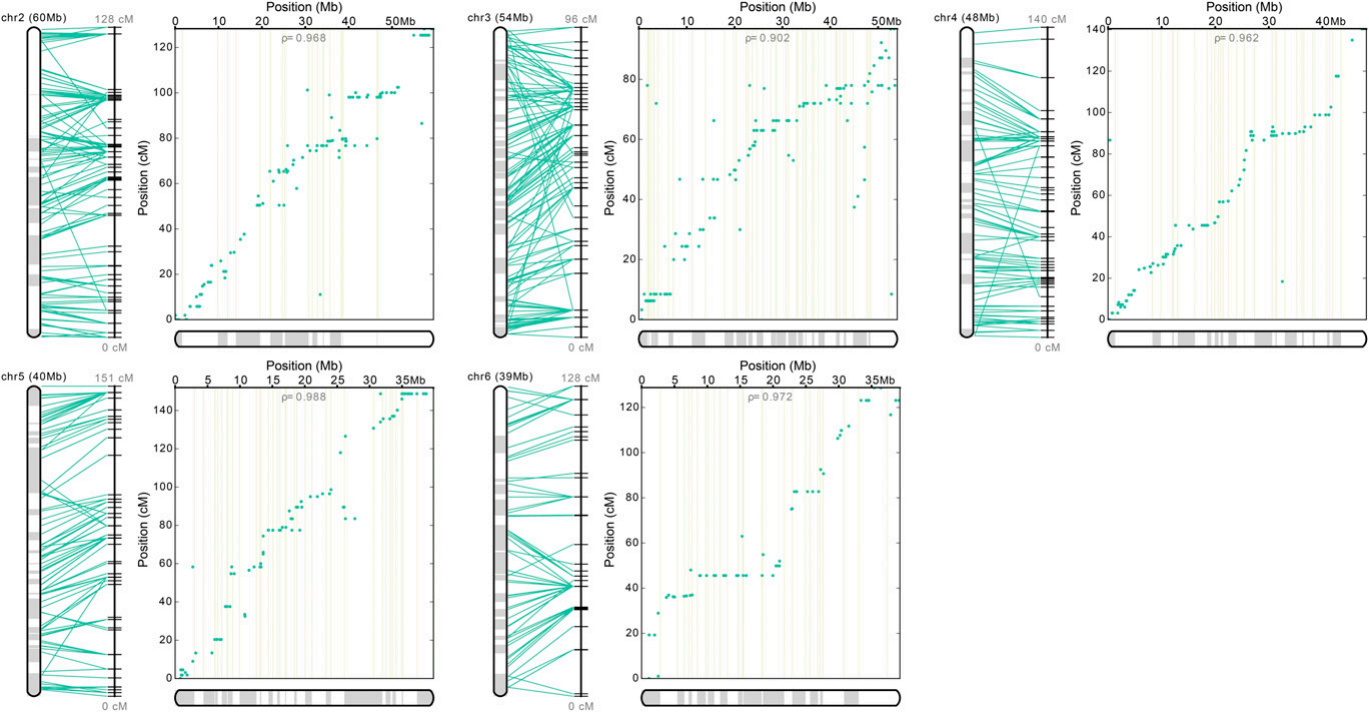
Superscaffolded map integrating linkage information from all chromosomes. For each linkage group the *y*-axis shows the position of each marker and its corresponding scaffold in the linkage map in centimorgans. On the *x*-axis is the subsequent position of each scaffold in megabase pairs after superscaffold assembly. The alternating gray and white bars represent contiguous scaffolds. Each linkage group is connected to its subsequent chromosome with green horizontal lines which denote SNP positions in the linkage group and its subsequent placement in the superscaffold assembly. Crossing of green horizontal lines indicate points of conflict where the linkage position and superscaffold position are not linear. The ratio between the longest monotonic subsequence and the total number of markers in the linkage group (ρ) indicate high collinearity between SNP marker position and scaffold placement.

A chromosome-scale assembly integrating linkage map information with whole-genome scaffold assembly information showed high agreement between position on the linkage map and position in the superscaffold whole-genome assembly (Figure 7). The accuracy of the chromosome-scale assemblies, as determined by the ratio between the longest monotonic subsequence and the total number of markers, was high for all chromosomes (ρ = 0.90–0.99). Using this method, 64.6% of the genome was placed in the chromosome assembly, and 52.8% of the scaffolds that were placed in the chromosome assembly were oriented with high confidence. In the dot plots for each chromosome, markers which share the same cM position on the linkage map while spaced apart on the physical scaffolds appear as a string of horizontal dots. These are areas of potentially low recombination that can be attributed to heterochromatic regions or structural polymorphisms, such as chromosomal inversions.

**Figure 8:**
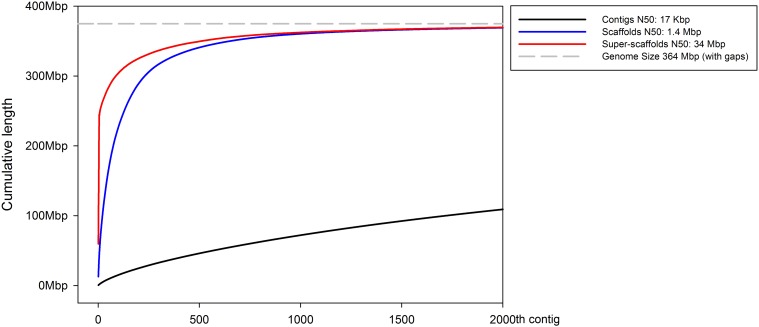
Cumulative assembly length. The cumulative lengths of the ALLPATHS-LG contig assembly (black), ALLPATHS-LG scaffold assembly (blue), and the ALLMAPS superscaffold assembly (red) show improvements in total assembly contiguity through this assembly improvement process.

An N50 of 34 Mb is reported for the superscaffolded whole-genome assembly, which improves upon the scaffold N50 of 1.4 Mb from the initial whole-genome assembly (Figure 8). A comparison between the melon fly superscaffold assembly and assemblies for other arthropods shows that it is among the most contiguous (Figure S3). The five chromosomes in the superscaffolded assembly gene set include 1885 out of 2622 (72%) complete single-copy arthropod-specific BUSCOs found in the scaffold assembly gene set, which leaves 737 complete single-copy arthropod-specific BUSCOs in scaffolds that are unplaced in the superscaffolded assembly. Out of 12,655 total unique genes in the *B. cucurbitae* scaffold assembly gene set, 9097 genes (72%) were in the superscaffold assembly. This shows there is a slight bias in the segments of genome included in the superscaffolds *vs.* unplaced scaffolds in terms of whether they contain coding sequences, as ∼64.6% of the assembly length is in superscaffolded chromosomes, which contain >70% of the annotated coding genes.

**Figure 9:**
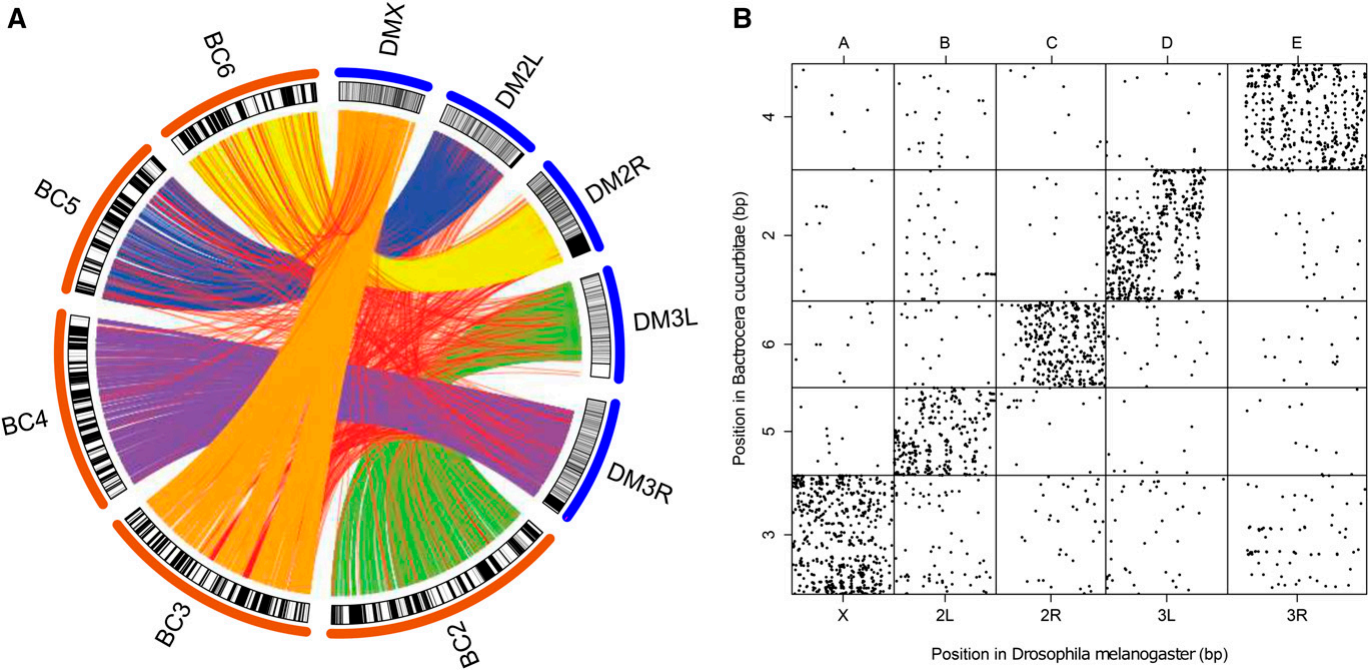
Synteny between *B. cucurbitae* and *D. melanogaster*. (A) An analysis for synteny shows how *B. cucurbitae* (BC) chromosomes correspond to the chromosome arms of *D. melanogaster* (DM) and the strong conservation of orthologous genes to Muller elements. Orange lines represent orthologous genes between their position on DM chromosome X and their position on BC chromosome 3, blue lines are between DM 2L and BC 5, yellow lines are between DM 2R and BC 6, green lines are between DM 3L and BC 2, purple lines are between DM 3R and BC 4, and red lines are connections between orthologous genes that are not on homologous chromosomes. (B) A dot plot, ordered by Muller element, showing the relationship between gene position in *D. melanogaster* and the position of its ortholog in *B. cucurbitae*. A linear regression analysis shows that there is no relationship between the position of an ortholog in a specific *D. melanogaster* Muller element and the corresponding position of this ortholog in the orthologous *B. cucurbitae* Muller element, which serves as evidence for widespread intrachromosomal translocations.

An analysis for synteny identified the homologous chromosomes of *B. cucurbitae* in *D. melanogaster*. The five chromosomes in *B. cucurbitae* that were identified through linkage mapping and superscaffolding correspond to the chromosome arms of *D. melanogaster* in a 1:1 ratio (Figure 9A). The *D. melanogaster* chromosome X and chromosome arms 2L, 2R, 3L, and 3R are Muller elements A–E respectively, and the genes within these Muller elements have been shown to be conserved within some species of *Drosophila* (Muller 1940; Sturtevant and Novitski 1941; Schaeffer *et al.* 2008). Through this analysis, 6117 single-copy orthologs were identified between mapped genes in *B. cucurbitae* and *D. melanogaster*. Of these single-copy orthologs, 90.6% were found on homologous chromosomes. For genes exhibiting collinearity between *B. cucurbitae* and *D. melanogaster*, 303 collinear blocks comprised of a total of 2582 genes were identified. Of these genes, 97% were on homologous chromosomes (Figure S6). This indicates a high level of conservation of gene composition on a chromosome level across the entirety of the genome between *D. melanogaster* and *B. cucurbitae*, which diverged between 68 and 148 million years ago (Junqueira *et al.* 2016; Hedges *et al.* 2015; Rainford *et al.* 2014; Kumar and Hedges 2011; Hedges *et al.* 2006). This pattern has been previously demonstrated between *D. melanogaster* and 11 other *Drosophila* species that showed high conservation of Muller elements, through karyotyping and synteny analysis (Schaeffer *et al.* 2008). This phenomenon has also been demonstrated in *B. tryoni*, in which 2030 genes were identified on the *B. tryoni* chromosome assembly, 93% of which were on homologous *Drosophila* Muller elements (Sved *et al.* 2016). Despite this gene conservation within a chromosome, the actual location of genes within a chromosome does not appear to be well conserved between *Bactrocera* and *Drosophila* species, with a high level of intrachromosomal translocations and gene shuffling (Figure 9B and Table S4).

In this study, all autosomes were identified through linkage mapping except X-linked and Y-linked scaffolds, which have remained unplaced and are a focus of future work. In previous studies, X-linked scaffolds have been shown to correspond with Muller element F in other *Bactrocera* (Vicoso and Bachtrog 2013; Sved *et al.* 2016). In these previous experiments, chromosome X was found by identifying orthologs of genes in *Drosophila* Muller element F in *B. oleae* and *B. tryoni*, and using read depth and heterozygosity respectively to infer if Muller element F genes were on chromosome X in both species. If this is the case, the abundance of sequences for genes orthologous to genes on *Drosophila* Muller element F should be half that of genes on the *Bactrocera* autosomes, and heterozygosity of these reads should be significantly lower or close to zero as well. In addition to the identification of the X chromosome in *B. cucurbitae* and other Tephritidae, the identification of Y will also be necessary for the generation of novel GSS in new tephritid species. With a goal of understanding the genetic sexing system in this species, and potentially transferring to other important pests, future work will be necessary to characterize the reciprocal autosome-Y translocation and their breakpoints in the GSS of *B. cucurbitae*, *B. dorsalis*, *C. capitata*, and *A. ludens*. In addition to understanding the mutations causing phenotypic sexing traits, recreating the translocation is required to make it sex-linked. The identification of DNA sequence motifs that result in stable nonhomologous end-joining after a double-stranded break could be performed, and these motifs targeted with CRISPR to generated targeted translocations for GSS development (Chen *et al.* 2015).

The QTL identified for white pupae in this study enabled the location of *wp*, which has been accurately placed on Muller element A (chromosome 3 in *B. cucurbitae*). Its location on chromosome 3 is at ∼78 cM in the linkage map, appropriately located near the tip of this chromosome, whose length is 97 cM. From this study, stable white and brown lines were derived from the F4 population and are maintained in colony. These stable lines will be the foundation for future work in identifying the causative mutation and gene for the white pupae phenotype. As in previous studies which identified candidate genes that govern traits of interest (Kloosterman *et al.* 2010; Ahsan *et al.* 2013; Gardner *et al.* 2016), whole-genome resequencing and RNA-seq analysis of individuals from this common genetic background segregating for white and wild-type brown pupae will facilitate the characterization of additional candidate genes and mutations involved in the phenotype.

Currently existing mass-produced SIT strains, such as the Vienna-7 and Vienna-8 strain of *C. capitata* and the Tapachula-7 strain of *A. ludens*, were created through ethyl methanesulfonate treatment, which results in random mutagenesis (Franz 2005; Zepeda-Cisneros *et al.* 2014). This not only generates desired mutations such as pupal color variants and temperature sensitivity, but also additional potentially detrimental mutations throughout the entirety of the genome. Future work in developing classical GSS-based SIT strains will require targeted mutagenesis, which can be achieved using transgenic techniques such as piggyBac transformation, which has been previously demonstrated in *C. capitata* and *A. suspensa* (Fraser *et al.* 1996; Handler *et al.* 1998; Handler and Harrell 2001), and CRISPR techniques, which are currently being explored in Tephritidae (Wang *et al.* 2013; Hwang *et al.* 2013; Friedland *et al.* 2013; Bassett and Liu 2014). With the identification of orthologous genes between *B. cucurbitae* and *D. melanogaster*, the entire suite of conditional lethal genes that have been identified in *D. melanogaster* are now available for use as targets for targeted mutagenesis, to create a conditional embryonic lethal GSS for *B. cucurbitae*. The *B. cucurbitae* genome and gene set have been included in the online web tool CHOPCHOP v.2 (Labun *et al.* 2016; Montague *et al.* 2014) to serve as a resource for CRISPR guide RNA design. Overall, this results in a strong foundation serving as a resource for functional genomic studies in this previously understudied species and other tephritid fruit flies.

### Conclusions

Here, we present an integrative approach using emerging genome sequencing technologies with classical genetics to characterize *wp* and assemble a high-quality, high-contiguity draft genome. In addition, QTL analysis showed that *wp* is located on chromosome 3 in *B. cucurbitae* (Muller element A, Drosophila X). Through further interrogation and SNP genotyping of tightly linked loci using a SNP assay, we found that the genotype of an individual fly can be inferred for the pupal color phenotype, suggesting close proximity of these loci to the actual causative mutation.

The availability of high-quality genomic resources necessary for future work in the development of SIT programs of new pest species, including placement of the assembly into a chromosomal context, and comparative genomics with other species to utilize the wealth of information in model insect genome systems. This study is unique in its thorough genomic and genetic characterization of a GSS. It was accomplished in a nonmodel insect system with economic importance, and a limiting amount of genomic DNA. The techniques used in this study can be a viable approach to assemble a genome at a chromosomal scale for other small insect systems. The outcome of this study is a foundational genomic tool set for *B. cucurbitae*, which will be used to facilitate its development as an SIT strain and reinforces the use of GSSs in SIT programs as a classic example of how the study of genetics can be implemented in pest management systems.

## Supplementary Material

Supplemental material is available online at www.g3journal.org/lookup/suppl/doi:10.1534/g3.117.040170/-/DC1.
