# Supplementary figures and images for: A Chromosome-Scale Assembly of the Bactrocera cucurbitae Genome Provides Insight to the Genetic Basis of white pupae
Source: G3 (Bethesda). 2017 Apr 20;7(6):1927–40. doi: 10.1534/g3.117.040170 (PMC5473769; doi:10.1534/g3.117.040170)

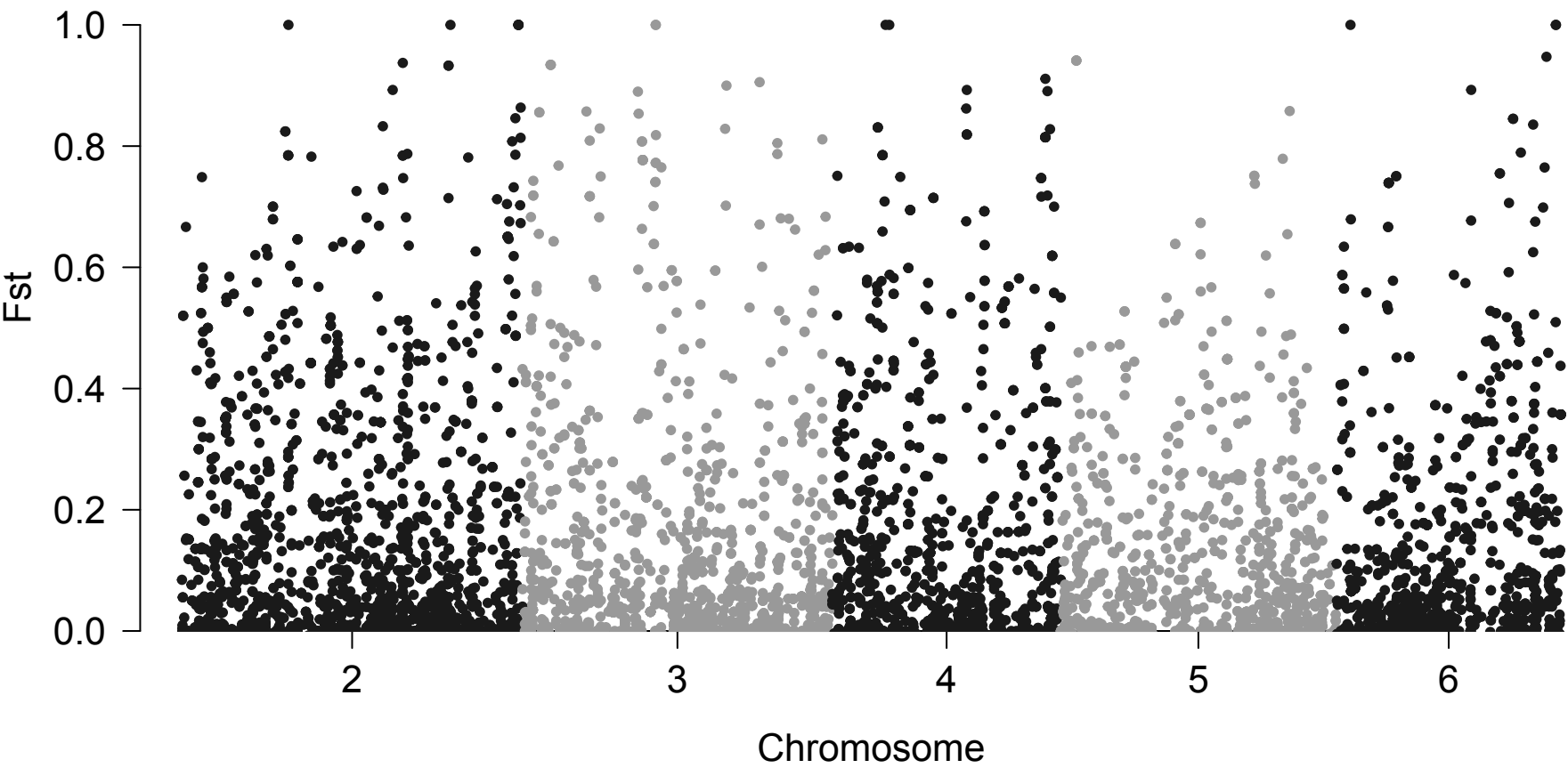

Supplement: Supplementary file 1 [file 1927FigureS1.pdf]

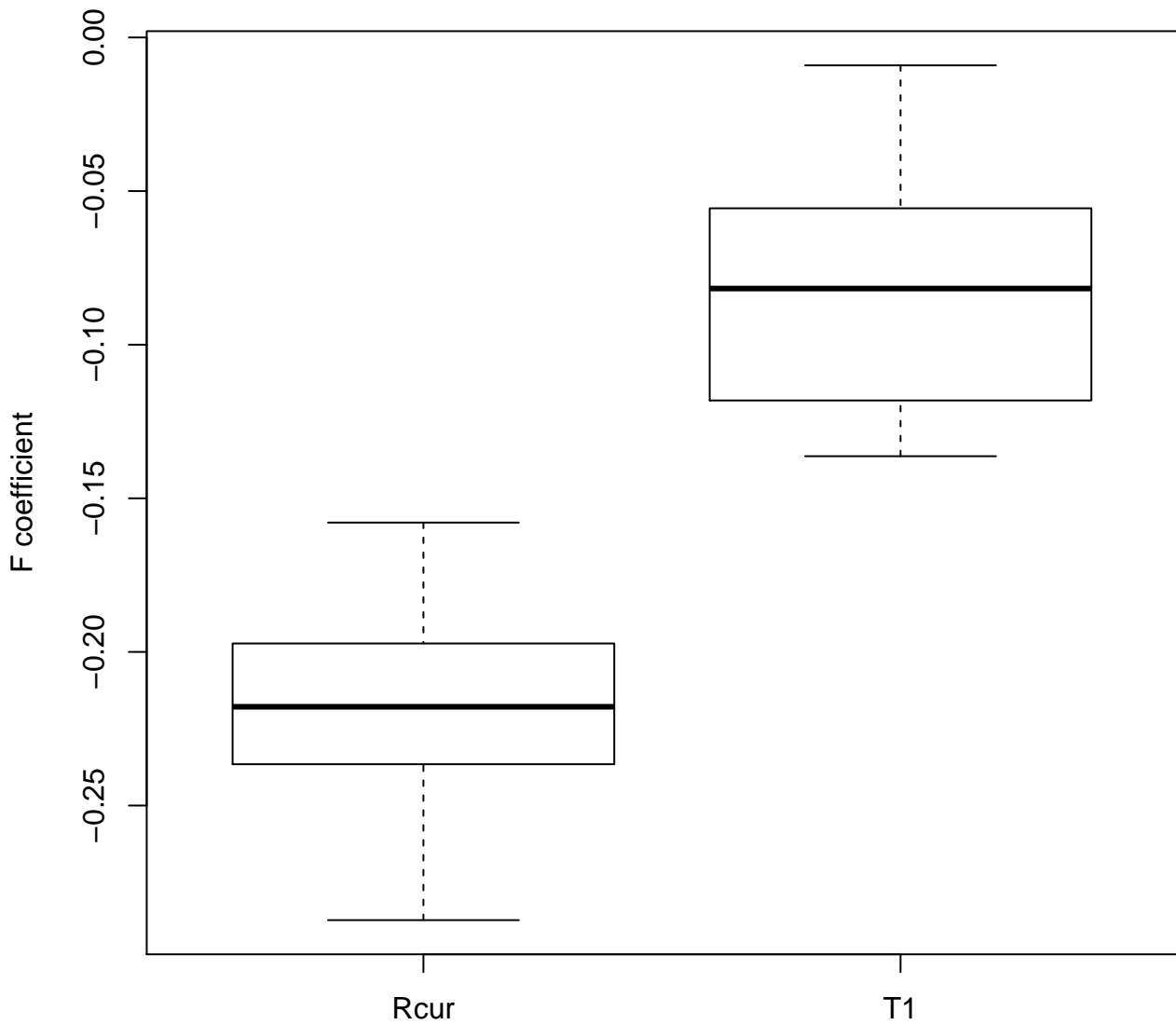

Supplement: Supplementary file 2 [file 1927FigureS2.pdf]

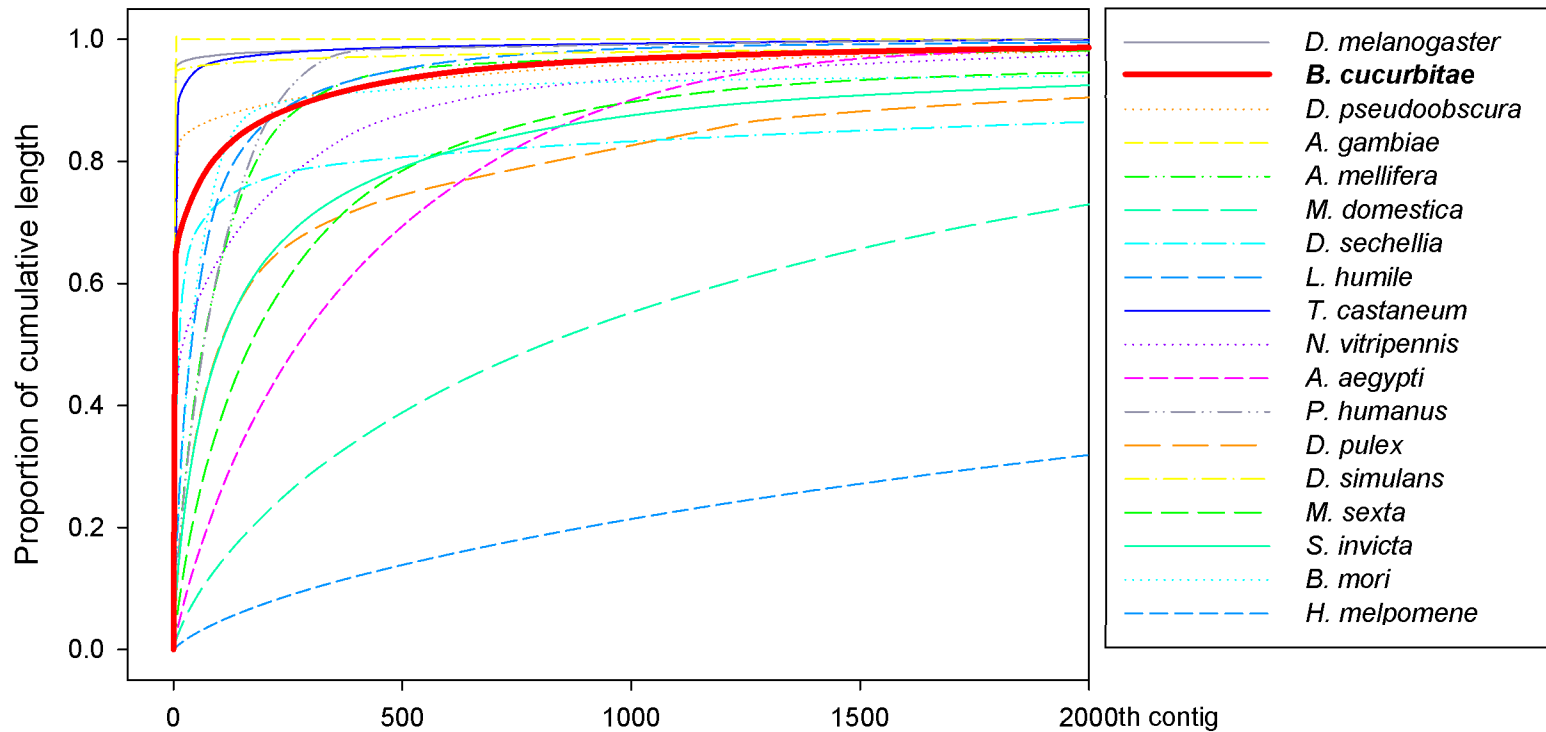

Supplement: Supplementary file 3 [file 1927FigureS3.pdf]

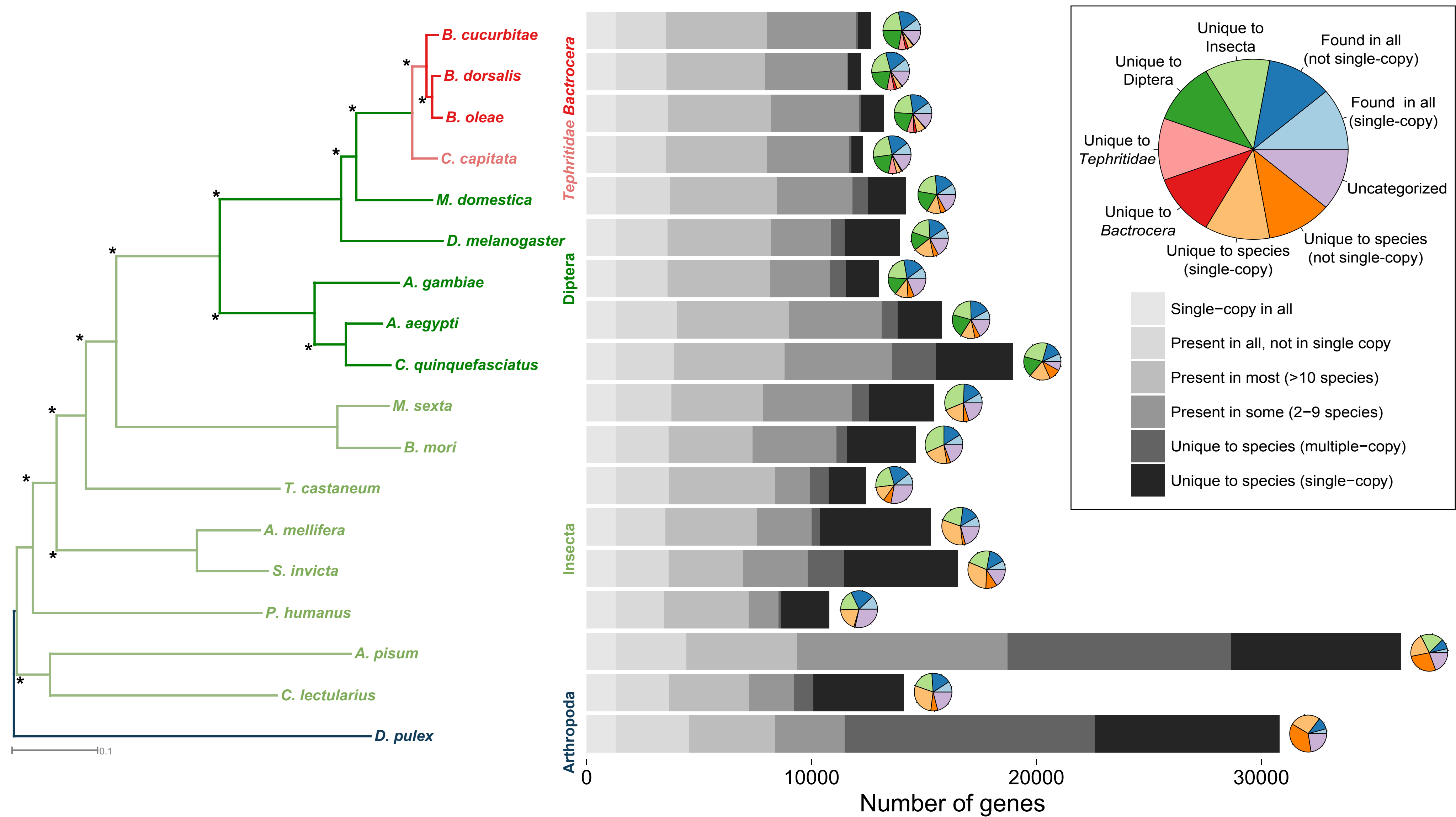

Supplement: Supplementary file 4 [file 1927FigureS4.pdf]

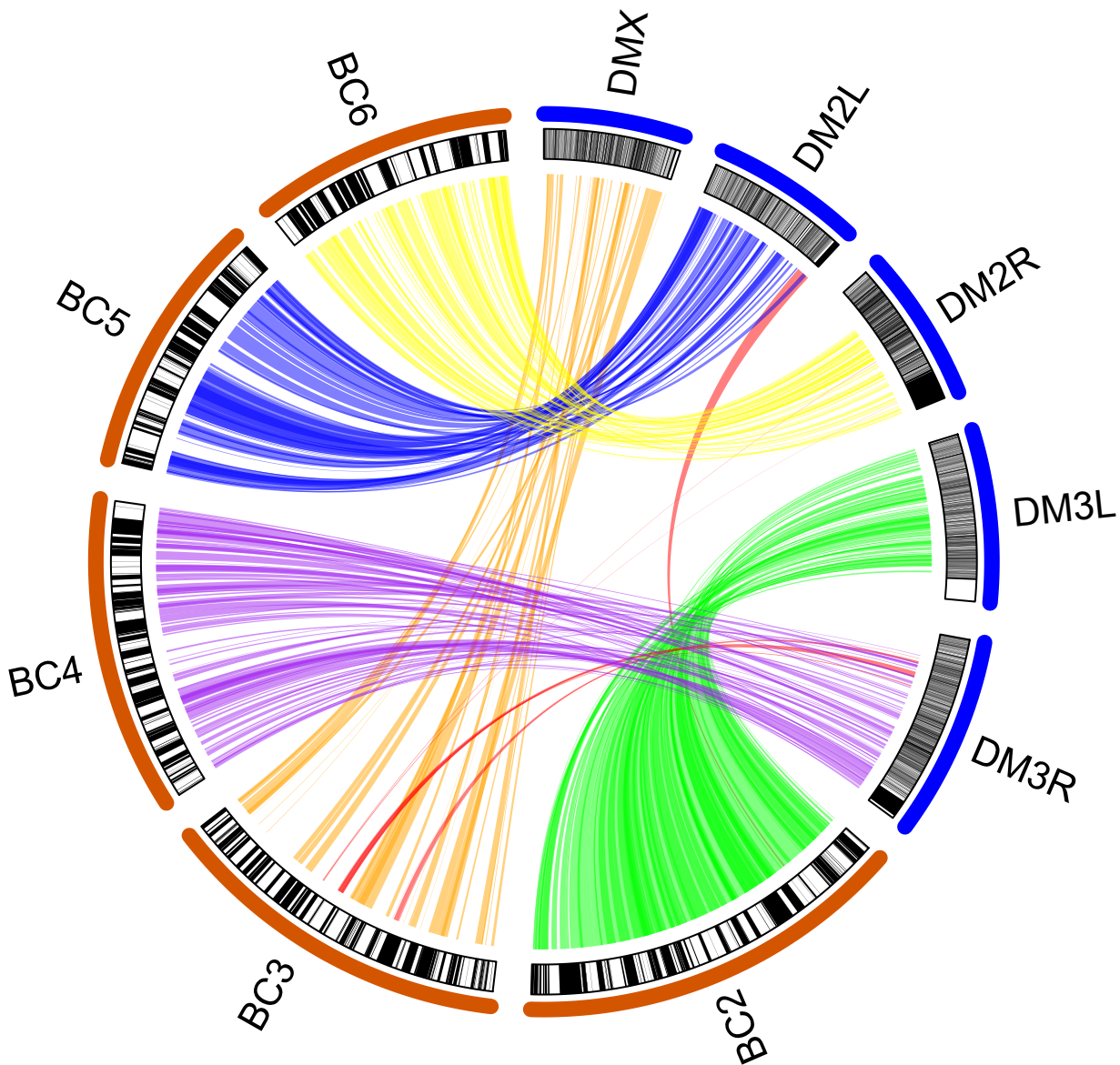

Supplement: Supplementary file 6 [file 1927FigureS6.pdf]
