# Supplementary material for: A Chromosome-Scale Assembly of the Bactrocera cucurbitae Genome Provides Insight to the Genetic Basis of white pupae
Source: G3 (Bethesda). 2017 Apr 20;7(6):1927–40. doi: 10.1534/g3.117.040170 (PMC5473769; doi:10.1534/g3.117.040170)
Supplement: Supplementary file 7 [file 1927FileS1.pdf]

Text S1

Orthology and phylogenetic context. The RefSeq gene annotation set was used to define orthologous genes between *B. cucurbitae* and other important arthropod genomes, and single copy orthologous proteins were used to perform a phylogenetic analysis to place *B. cucurbitae* in context among other arthropods. The set of arthropods used in this analysis included two other species in the genus *Bactrocera*: *B. dorsalis*, *B. oleae*; an additional member of Tephritidae: *C. capitata*; five additional dipterans: *M. domestica*, *D. melanogaster*, *Anopheles gambiae*, *Aedes aegypti*, and *Culex quinquefasciatus*; eight additional insects: *Menduca sexta*, *Bombyx mori*, *Tribolium castaneum*, *Apis mellifera*, *Solenopsis invicta*, *Pediculus humanus*, *Acyrthosiphon pisum*, and *Cimex lectularius*; and one non-insect arthropod, *Daphnia pulex*, as an outgroup. For each of these species, the most recent gene annotation set was downloaded from their respective databases (Table S1). The peptide sequence corresponding to the longest transcript for each species was extracted using custom scripts and arthropod BUSCOs were identified with BUSCO v1.1b1. The resulting 2,591 single-copy orthologs were extracted from their respective peptide files, aligned with muscle (Edgar, 2004a,b), trimmed with trimAL using the options "-w 3 -gt 0.95 -st 0.01" (Capella-Gutierrez et al., 2009), concatenated, and formatted to a phylip format using the program ElConcatenero (Silva, 2014). The phylogenetic relationship of these species was determined using RAxML (Stamatakis et al., 2005; Stamatakis, 2014) using the PROTGAMMA amino acid substitution model, a model which has been demonstrated to define a phylogenetic tree topology with a higher likelihood than alternative amino acid substitution models (Le and Gascuel, 2008), and 1000 bootstrap replicates. The resulting tree was visualized using Dendroscope 3.2.10 (Huson and Scornavacca, 2012). Orthologous genes were identified by performing an all-by-all BLASTp search for each complete peptide sequence file for each species. OrthoMCL and the MCL clustering algorithm (Van Dongen, 2000) were used to predict orthologous groups from the BLAST results, and orthologs, co-orthologs, and inparalogs were defined between all proteins from all species. The number of orthologous groups unique to species and shared between some, most, and all species in the data set were summarized in addition to orthologous groups found to be shared within the genus *Bactrocera*, family *Tephritidae*, order Diptera, and class Insecta. Cumulative bar plots and pie charts were visualized using R version 3.2.2 (R Core Team, 2015). Supplemental data supporting orthology analysis is available online at the USDA Ag Data Commons (`https://data.nal.usda.gov`) under DOI 10.15482/USDA.ADC/1329913.
