# Supplementary material for: A Chromosome-Scale Assembly of the Bactrocera cucurbitae Genome Provides Insight to the Genetic Basis of white pupae
Source: G3 (Bethesda). 2017 Apr 20;7(6):1927–40. doi: 10.1534/g3.117.040170 (PMC5473769; doi:10.1534/g3.117.040170)
Supplement: Supplementary file 8 [file 1927FileS2.pdf]

Text S2

Discrimination assay. The SNP locus displaying the strongest linkage to the *wp* phenotype as determined by a permutation test was located on Scaffold NW_011863770.1 at base position 174,324, on the super-scaffold associated with chromosome 3, syntenic with *Drosophila* chromosome X (Muller element A). There were several other SNP loci that showed positive association with the phenotype located close to this locus. To confirm the validity of this analysis, TaqMan SNP genotyping assays were designed around these markers (Thermo Scientific, Waltham, MA). Some adjascent SNPs with the high LOD scores failed to amplify due to variation in the sequences flanking the SNP, but the SNP at scaffold NW_011863770.1 at position 174,324 was consistent in discriminating between mutant white and wild-type brown pupae individuals. The qPCR amplifications used to run the discrimination assay were performed in volumes of $10.0\ \mu$L containing $5.0\ \mu$L SensiFAST^{TM} Hi-ROX Genotyping Kit (Bioline, London, UK), $0.5\ \mu$L 20x TaqMan SNP assay, $2.0\ \mu$L template DNA, and $2.5\ \mu$L Nuclease-free H_2O. The TaqMan SNP assay that was successful (Scaffold NW_011863770, position 174,324) was designed with the forward primer sequence 5'-CACTGGGACAGATGTCACAAGA-3', reverse primer sequence 5'-GGCTTCCAACAGCTTCATCTC-3, wild-type allele reporter (VIC) sequence 5'-CACTGCCTTCCATCGC-3', and mutant allele reporter (FAM) sequence 5'-ACTGCCTCCCATCGC-3'. The genotyping assay was performed on a StepOnePlus Real-Time PCR system (Thermo Scientific, Waltham, MA) at a fast ramp speed and under the following cycling conditions: initial pre-PCR read stage at 25.0°C for 30s; 95.0°C for 20s; 40 cycles of 95.0°C for 3s and 60°C for 20s; and a final post-PCR read stage at 25.0°C for 30.s. Data analysis on the change (Δ) in fluorescence for both reporters was performed on the StepOne Software v2.3 (Thermo Scientific, Waltham, MA). This assay was tested on individuals of known genotype from the T1 and wild-type laboratory colonies, as well as brown pupae individuals that were the subsequent progeny from the mapping populations. Utilizing this assay, stable homozygous brown and homozygous white lines were generated, by genotyping individuals in the F4 population, and setting up homozygous isocrosses. These lines will be maintained and used in subsequent follow-up studies further examining the *white pupae* mutation.
