# Supplementary material for: A Chromosome-Scale Assembly of the Bactrocera cucurbitae Genome Provides Insight to the Genetic Basis of white pupae
Source: G3 (Bethesda). 2017 Apr 20;7(6):1927–40. doi: 10.1534/g3.117.040170 (PMC5473769; doi:10.1534/g3.117.040170)
Supplement: Supplementary file 9 [file 1927FileS3.pdf]

Supplemental File S3: Supplemental material file descriptions and supplemental references

File S1 - Orthology and phylogenetic context. (.pdf)

File S2 - Discrimination assay. (.pdf)

File S3: Supplemental Material File Descriptions and references. (.pdf)

Table S1 - Data sources for species used in ortholog assignment. (.pdf)

Table S2 - ddRAD read statistics. (.pdf)

Table S3 - Candidate genes within significant QTL region. (.pdf)

Table S4 - Regression between position of gene orthologs in D. melanogaster and B. cucurbitae. (.pdf)

Figure S1- Manhattan plot showing genome-wide FST values Calculation of Weir-Cockeram's FST between the wild-type laboratory colony and the T1-Melon colony shows fixed differences where FST is equal to 1 across the genome. (.pdf)

Figure S2 - Genome-wide levels of heterozygosity. Calculation of heterozygosity in both colonies show significantly higher levels of heterozygosity in both colonies relative to the expectation based on Hardy-Weinberg equilibrium. This indicates these colonies are not experiencing inbreeding in colony. (.pdf)

Figure S3 - Comparison of cumulative assembly length with notable arthropod species. The cumulative lengths of the ALLMAPS super-scaffold assembly show the B. cucurbitae genome to be among the assemblies that have the highest contiguity shown by the steeper slope which indicates an assembly in a fewer number of large pieces. (.pdf)

Figure S4 - Orthology analysis between B. cucurbitae and other arthropod gene sets. The phylogenetic relationship of B. cucurbitae and 17 species in Arthropoda was estimated using a maximum likelihood analysis of single-copy orthologous protein sequences, 1000 bootstrap replicates, and rooted using D. pulex (methods are described in File S1). The scale bar shows the branch distance for 0.1 amino acid substitution per site and all nodes with a bootstrap value of 100 are noted with an asterisk. The gray horizontal bars for each species show the absolute number of proteins that are: single-copy orthologs in all species, present in all species not in single-copy, present in >10 of the species in the analysis, present in a 2-9 of the species in the analysis, and unique to the species. The pie charts at the end of the gray bars show the proportion of genes for each species that are unique to the species in single-copy, unique to the species not in single-copy, unique to the genus Bactrocera, unique to Tephritidae, unique to Diptera, unique to insecta, found in all species in the analysis in single-copy, and found in all species in the analysis not in single-copy. A total of 1,299 single copy orthologs were found in all species. In the B. curubitae gene set, 162 orthologs were found to be unique to the three Bactrocera in the dataset, 1,088 were unique to the four Tephritidae, 3,597 were unique to the nine Diptera, and 5,596 were unique to the 17 Arthropoda. (.pdf)

Figure S5 - Allelic discrimination plot. Control individuals of known phenotypes were genotyped using a Taq-Man SNP genotyping assay designed for the highest scoring QTL locus (methods are described in File S2). The observed genotypes for the white pupae GSS females, the brown pupae GSS males, and brown pupae wild males and females segregated with the white pupae phenotype as predicted. (.eps)

Figure S6 - Synteny plot for collinear orthologs. An analysis for synteny for orthologous genes found in collinear blocks between B. cucurbitae (BC) and D. melanogaster (DM) shows that a higher proportion of genes are found in homologous chromosomes in contrast to the same analysis performed on all orthologous genes. (.pdf)

Supplemental References

Adams, M. D., S. E. Celniker, R. A. Holt, C. A. Evans, J. D. Gocayne et al., 2000 The genome sequence of Drosophila melanogaster. Science 287: 2185-2195.

Attrill, H., K. Falls, J. L. Goodman, G. H. Millburn, G. Antonazzo et al., 2016 FlyBase: establishing a Gene Group resource for Drosophila melanogaster. Nucleic Acids Research 44: D786-D792.

Capella-Gutierrez, S., J. M. Silla-Martinez, and T. Gabaldon, 2009 trimAl: a tool for automated alignment trimming in large-scale phylogenetic analyses. Bioinformatics 25: 1972-1973.

Colbourne, J. K., M. E. Pfrender, D. Gilbert, W. K. Thomas, A. Tucker et al., 2011 The Ecoresponsive Genome of Daphnia pulex. Science 331: 555-561.

Drosopoulou, E., I. Nakou, and P. Mavragani-Tsipidou, 2014 The Bactrocera oleae genome: localization of nine genes on the polytene chromosomes of the olive fruit fly (Diptera: Tephritidae). Genome 57: 573-576.

Edgar, R. C., 2004a MUSCLE: a multiple sequence alignment method with reduced time and space complexity. Bmc Bioinformatics 5: 1-19.

Edgar, R. C., 2004b MUSCLE: multiple sequence alignment with high accuracy and high throughput. Nucleic Acids Research 32: 1792-1797.

Elsik, C. G., K. C. Worley, A. K. Bennett, M. Beye, F. Camara et al., 2014 Finding the missing honey bee genes: lessons learned from a genome upgrade. BMC Genomics 15:.

Giraldo-Calderon, G. I., S. J. Emrich, R. M. MacCallum, G. Maslen, E. Dialynas et al., 2015 VectorBase: an updated bioinformatics resource for invertebrate vectors and other organisms related with human diseases. Nucleic Acids Research 43: D707-D713.

Holt, R. A., G. M. Subramanian, A. Halpern, G. G. Sutton, R. Charlab et al., 2002 The genome sequence of the malaria mosquito Anopheles gambiae. Science 298: 129-+.

Huson, D. H., and C. Scornavacca, 2012 Dendroscope 3: An Interactive Tool for Rooted Phylogenetic Trees and Networks. Systematic Biology 61: 1061-1067.

Kersey, P. J., J. E. Allen, I. Armean, S. Boddu, B. J. Bolt et al., 2016 Ensembl Genomes 2016: more genomes, more complexity. Nucleic Acids Research 44: D574-D580.

Le, S. Q., and O. Gascuel, 2008 An Improved General Amino Acid Replacement Matrix. Molecular Biology and Evolution 25: 1307-1320.

Legeai, F., S. Shigenobu, J.-P. Gauthier, J. Colbourne, C. Rispe et al., 2010 AphidBase: A centralized bioinformatic resource for annotation of the pea aphid genome. Insect molecular biology 19: 5-12.

Mita, K., M. Kasahara, S. Sasaki, Y. Nagayasu, T. Yamada et al., 2004 The genome sequence of silkworm, Bombyx mori. DNA Research 11: 27-35.

Munoz-Torres, M. C., J. T. Reese, C. P. Childers, A. K. Bennett, J. P. Sundaram et al., 2011 Hymenoptera Genome Database: integrated community resources for insect species of the order Hymenoptera. Nucleic Acids Research 39: D658-D662.

Nene, V., J. R. Wortman, D. Lawson, B. Haas, C. Kodira et al., 2007 Genome sequence of Aedes aegypti, a major arbovirus vector. Science 316: 1718-1723.

Nordberg, H., M. Cantor, S. Dusheyko, S. Hua, A. Poliakov et al., 2014 The genome portal of the Department of Energy Joint Genome Institute: 2014 updates. Nucleic Acids Research 42: D26-D31.

O'Leary, N. A., M. W. Wright, J. R. Brister, S. Ciufo, D. Haddad et al., 2016 Reference sequence (RefSeq) database at NCBI: current status, taxonomic expansion, and functional annotation. Nucleic Acids Res 44: D733-45.

Papanicolaou, A., M. F. Schetelig, P. Arensburger, P. W. Atkinson, J. B. Benoit et al., 2016 The whole genome sequence of the Mediterranean fruit fly, Ceratitis capitata (Wiedemann), reveals insights into the biology and adaptive evolution of a highly invasive pest species. Genome Biology 17: 192.

Pittendrigh, B. R., J. M. Clark, J. S. Johnston, S. H. Lee, J. Romero-Severson et al., 2006 Sequencing of a new target genome: the Pediculus humanus humanus (Phthiraptera: Pediculidae) genome project. Journal of Medical Entomology 43: 1103-1111.

Poelchau, M., C. Childers, G. Moore, V. Tsavatapalli, J. Evans et al., 2015 The i5k Workspace@NAL-enabling genomic data access, visualization and curation of arthropod genomes. Nucleic Acids Research 43: D714-D719.

R Core Team, 2015 R: A Language and Environment for Statistical Computing. R Foundation for Statistical Computing, Vienna, Austria.

Richards, S., R. A. Gibbs, N. M. Gerardo, N. Moran, A. Nakabachi et al., 2010 Genome Sequence of the Pea Aphid Acyrthosiphon pisum. Plos Biology 8:.

Richards, S., R. A. Gibbs, G. M. Weinstock, S. J. Brown, R. Denell et al., 2008 The genome of the model beetle and pest Tribolium castaneum. Nature 452: 949-955.

Rosenfeld, J. A., D. Reeves, M. R. Brugler, A. Narechania, S. Simon et al., 2016 Genome assembly and geospatial phylogenomics of the bed bug Cimex lectularius. Nature Communications 7:.

Scott, J. G., W. C. Warren, L. W. Beukeboom, D. Bopp, A. G. Clark et al., 2014 Genome of the house fly, Musca domestica L., a global vector of diseases with adaptations to a septic environment. Genome Biology 15:.

Silva, D. 2014. El Concatenero. GitHub repository, https://github.com/ODiogoSilva/ElConcatenero

Stamatakis, A., 2014 RAxML version 8: a tool for phylogenetic analysis and post-analysis of large phylogenies. Bioinformatics 30: 1312-1313.

Stamatakis, A., T. Ludwig, and H. Meier, 2005 RAxML-III: a fast program for maximum likelihood-based inference of large phylogenetic trees. Bioinformatics 21: 456-463.

Van Dongen, S., 2000 Graph clustering by flow simulation [Thesis]: University of Utrecht, Uterecht.

Weinstock, G. M., G. E. Robinson, R. A. Gibbs, K. C. Worley, J. D. Evans et al., 2006 Insights into social insects from the genome of the honeybee Apis mellifera. Nature 443: 931-949.

Wurm, Y., J. Wang, O. Riba-Grognuz, M. Corona, S. Nygaard et al., 2011 The genome of the fire ant Solenopsis invicta. Proceedings of the National Academy of Sciences of the United States of America 108: 5679-5684.
