# Supplementary material for: A Chromosome-Scale Assembly of the Bactrocera cucurbitae Genome Provides Insight to the Genetic Basis of white pupae
Source: G3 (Bethesda). 2017 Apr 20;7(6):1927–40. doi: 10.1534/g3.117.040170 (PMC5473769; doi:10.1534/g3.117.040170)
Supplement: Supplementary file 10 [file 1927TableS1.pdf]

**Table S1. Data sources for species used in phylogenetic analyses.**

| Species | Version | Source |
|---|---|---|
| *B. dorsalis* | 1.1 | ftp://ftp.ncbi.nlm.nih.gov/genomes/refseq/ |
| *B. oleae* | 2.0 | https://i5k.nal.usda.gov |
| *C. capitata* | 1.1 | ftp://ftp.ncbi.nlm.nih.gov/genomes/refseq/ |
| *M. domestica* | 1.1 | https://www.vectorbase.org/ |
| *D. melanogaster* | 6.08 | http://flybase.org/ |
| *A. gambiae* | 4.3 | https://www.vectorbase.org/ |
| *A. aegypti* | 3.3 | https://www.vectorbase.org/ |
| *C. quinquefasciatus* | 2.2 | https://www.vectorbase.org/ |
| *M. sexta* | 1.0 | https://i5k.nal.usda.gov |
| *B. mori* | 1.29 | http://metazoa.ensembl.org/ |
| *T. castaneum* | 3.0 | https://i5k.nal.usda.gov |
| *A. mellifera* | 3.2 | http://hymenopteragenome.org/beebase/ |
| *S. invicta* | 2.2.3 | http://hymenopteragenome.org/solenopsis/ |
| *P. humanus* | 2.1 | https://www.vectorbase.org/ |
| *A. pisum* | 2.1b | http://bipaa.genouest.org/is/aphidbase/ |
| *C. lectularius* | 1.2 | https://i5k.nal.usda.gov |
| *D. pulex* | 1.1 | http://genome.jgi.doe.gov/ |
