# Supplementary material for: A Chromosome-Scale Assembly of the Bactrocera cucurbitae Genome Provides Insight to the Genetic Basis of white pupae
Source: G3 (Bethesda). 2017 Apr 20;7(6):1927–40. doi: 10.1534/g3.117.040170 (PMC5473769; doi:10.1534/g3.117.040170)
Supplement: Supplementary file 11 [file 1927TableS2.pdf]

Table S2. Read depth for each individual in ddRAD sequencing library.

Generation	ID	Total reads	No RAD	Low quality	Retained
F2	BF_6	257241	1034	81	256126
F2	BM_6	459646	2363	167	457116
F3	BF_6_6	920937	873	329	919735
F3	BM_6_6	577261	238	213	576810
F4	BF_6_6_2	992869	2462	365	990042
F4	BF_6_6_15	567745	2056	222	565467
F4	BF_6_8_7	619941	980	226	618735
F4	BF_6_6_17	390532	1190	133	389209
F4	BF_6_6_19	658329	174	234	657921
F4	BF_6_6_20	587035	1305	225	585505
F4	BF_6_6_29	726608	327	254	726027
F4	BF_6_6_31	675918	978	268	674672
F4	BM_6_6_26	810476	2135	308	808033
F4	BF_6_6_23	336018	2166	144	333708
F4	BM_6_6_6	285099	8850	90	276159
F4	BF_6_6_18	464788	617	154	464017
F4	BF_6_6_30	1008677	2417	360	1005900
F4	BM_6_6_3	271463	958	101	270404
F4	BF_6_6_34	635728	459	239	635030
F4	BM_6_6_25	455262	185	176	454901
F4	BF_6_6_22	712681	1118	257	711306
F4	BM_6_6_24	664314	1595	256	662463
F4	BF_6_6_1	867266	2173	337	864756
F4	BM_6_6_11	421880	1978	182	419720
F4	BF_6_6_33	707791	1926	245	705620
F4	BM_6_6_1	719852	191	271	719390
F4	BM_6_6_22	398612	113	128	398371
F4	BM_6_6_5	598864	265	203	598396
F4	BM_6_6_21	1049830	576	397	1048857
F4	BF_6_6_32	271851	3459	93	268299
F4	BM_6_6_4	506370	173	193	506004
F4	WF_6_6_1	1193211	1266	466	1191479
F4	WF_6_6_9	879730	5618	332	873780
F4	WM_6_6_2	549573	762	203	548608
F4	WM_6_6_1	852240	6207	318	845715
F4	WM_6_6_5	1148930	4070	431	1144429
F4	WF_6_6_6	1335541	1118	517	1333906
F4	WM_6_6_3	1498731	443	506	1497782
F4	WM_6_6_4	991419	1579	375	989465
F4	WF_6_6_8	754284	2263	280	751741

Generation	ID	Total reads	No RAD	Low quality	Retained
F3	BF_6_8	585002	696	204	584102
F3	BM_6_8	152780	2114	55	150611
F4	BM_6_8_12	233556	372	84	233100
F4	BM_6_8_22	586604	1877	246	584481
F4	BF_6_8_1	1063928	2240	369	1061319
F4	BF_6_8_3	660497	1969	220	658308
F4	BF_6_8_4	313104	102	101	312901
F4	BM_6_8_2	171179	290	58	170831
F4	BM_6_8_3	169426	195	53	169178
F4	BM_6_8_17	819747	569	314	818864
F4	BM_6_8_19	608368	1604	222	606542
F4	BM_6_8_20	671369	2277	253	668839
F4	BM_6_8_21	736391	2174	318	733899
F4	BM_6_8_4	233806	2576	79	231151
F4	BM_6_8_15	123901	1023	41	122837
F4	BM_6_8_18	537384	1368	199	535817
F4	BF_6_8_2	271504	1329	85	270090
F4	BM_6_8_1	400589	208	164	400217
F4	BM_6_8_7	286677	1406	118	285153
F4	BM_6_8_11	498269	183	193	497893
F4	BM_6_8_8	506698	829	190	505679
F4	BM_6_8_13	787498	3065	318	784115
F4	WM_6_8_3	624567	2332	221	622014
F4	WM_6_8_4	764134	7676	262	756196
F4	WF_6_8_1	698859	938	284	697637
F4	WM_6_8_2	572046	3438	220	568388
F4	WM_6_8_5	998850	838	351	997661
F3	BF_6_16	601381	2975	210	598196
F3	BM_6_16	535117	581	234	534302
F4	BF_6_16_4	332553	213	127	332213
F4	BF_6_16_5	211715	135	72	211508
F4	BF_6_16_9	328991	1161	118	327712
F4	BM_6_16_1	523914	1678	208	522028
F4	BF_6_16_10	763222	1093	292	761837
F4	BM_6_16_4	667504	2624	247	664633
F4	BM_6_16_2	543718	2051	230	541437
F4	BM_6_16_3	516943	168	208	516567
F4	WF_6_16_2	648894	220	258	648416
F4	WM_6_16_2	869087	540	320	868227
F4	WM_6_16_3	667161	639	266	666256
F4	WF_6_16_3	665157	3644	249	661264

Generation	ID	Total reads	No RAD	Low quality	Retained
F3	BF_6_44	416334	3055	165	413114
F3	BM_6_44	1243359	1948	470	1240941
F4	BF_6_44_11	1207258	324	416	1206518
F4	BF_6_44_10	1056998	1157	405	1055436
F4	BM_6_44_4	794340	6835	311	787194
F4	BF_6_44_2	875330	1950	331	873049
F4	BF_6_44_4	890337	4494	343	885500
F4	BF_6_44_7	945891	5838	347	939706
F4	BF_6_44_12	610103	1020	219	608864
F4	BF_6_44_8	459633	262	192	459179
F4	BM_6_44_5	859808	2003	297	857508
F4	BM_6_44_12	710576	1827	262	708487
F4	BF_6_44_1	930144	797	347	929000
F4	BF_6_44_3	1028410	764	355	1027291
F4	BM_6_44_11	649238	4135	201	644902
F4	BF_6_44_6	1029054	2691	357	1026006
F4	BF_6_44_9	1109007	2817	398	1105792
F4	BM_6_44_7	640525	204	240	640081
F4	WF_6_44_10	1005502	546	383	1004573
F4	WM_6_44_1	1530925	906	645	1529374
F4	WM_6_44_3	1033978	742	412	1032824
F4	WM_6_44_5	861173	2732	286	858155
F4	WF_6_44_3	830991	2911	317	827763
F4	WF_6_44_4	870330	2670	327	867333
F4	WM_6_44_2	275383	4266	96	271021
F4	WF_6_44_1	167672	1905	50	165717
F4	WF_6_44_9	331384	1064	104	330216
F4	WF_6_44_6	138102	1057	41	137004
F4	WF_6_44_7	301320	240	102	300978
F4	WF_6_44_8	440633	737	156	439740
Parental line	RmelF_1	990384	4610	356	985418
Parental line	RmelF_3	1552116	3107	571	1548438
Parental line	RmelF_4	996939	2769	357	993813
Parental line	RmelF_8	1021512	484	408	1020620
Parental line	RmelF_2	1379093	3409	527	1375157
Parental line	RmelF_7	772443	304	291	771848
Parental line	RmelM_2	713330	1684	277	711369
Parental line	RmelM_3	1326194	4284	492	1321418
Parental line	RmelM_7	520617	3585	199	516833
Parental line	RmelM_8	492184	14295	203	477686
Parental line	RmelM_1	407504	1221	139	406144
Parental line	RmelM_4	557950	6361	213	551376

Generation	ID	Total reads	No RAD	Low quality	Retained
F3	BF_6_58	656302	1000	238	655064
F3	BM_6_58	424382	330	157	423895
F4	BF_6_58_8	333669	152	123	333394
F4	BF_6_58_9	234633	1035	70	233528
F4	BM_6_58_8	972221	4689	332	967200
F4	BF_6_58_3	882382	2973	328	879081
F4	BF_6_58_11	770250	7073	281	762896
F4	BF_6_58_15	855096	1410	323	853363
F4	BM_6_58_1	702028	4797	232	696999
F4	BM_6_58_2	1205662	1763	478	1203421
F4	BM_6_58_6	701172	620	252	700300
F4	BF_6_58_2	795618	673	303	794642
F4	BF_6_58_4	573935	1636	206	572093
F4	BF_6_58_5	768760	1693	284	766783
F4	BF_6_58_6	812962	2074	325	810563
F4	BF_6_58_7	492834	2761	187	489886
F4	BF_6_58_10	849751	1924	298	847529
F4	BF_6_58_14	266370	142	92	266136
F4	BF_6_58_17	483212	279	169	482764
F4	BM_6_58_12	465838	413	174	465251
F4	BF_6_58_1	679542	797	249	678496
F4	BF_6_58_12	831428	3734	278	827416
F4	BF_6_58_16	665238	245	265	664728
F4	BM_6_58_7	623998	574	235	623189
F4	BM_6_58_3	274549	5037	109	269403
F4	BM_6_58_4	708251	988	237	707026
F4	BM_6_58_9	679031	6084	270	672677
F4	BM_6_58_10	565473	4293	216	560964
F4	BM_6_58_11	722686	1077	240	721369
F4	WF_6_58_3	983546	361	385	982800
F4	WM_6_58_1	790121	1449	288	788384
F4	WM_6_58_3	571854	2557	213	569084
Parental line	T1F_3	1131896	1998	455	1129443
Parental line	T1F_6	938772	765	346	937661
Parental line	T1F_8	1598954	1474	540	1596940
Parental line	T1F_1	1874444	3732	691	1870021
Parental line	T1F_4	1444709	2718	521	1441470
Parental line	T1F_5	1208360	3994	437	1203929
Parental line	T1F_7	960353	9035	331	950987
Parental line	T1M_3	1624934	4019	577	1620338
Parental line	T1M_4	1215001	601	427	1213973
Parental line	T1M_1	1045652	392	392	1044868
Parental line	T1M_2	1315813	1169	473	1314171
