# Supplementary material for: A Chromosome-Scale Assembly of the Bactrocera cucurbitae Genome Provides Insight to the Genetic Basis of white pupae
Source: G3 (Bethesda). 2017 Apr 20;7(6):1927–40. doi: 10.1534/g3.117.040170 (PMC5473769; doi:10.1534/g3.117.040170)
Supplement: Supplementary file 12 [file 1927TableS3.pdf]

Table S3. Genes related to cuticle structure and sclerotization within region with significant LOD score

Gene ID	Gene product
LOC105210517	pupal cuticle protein 36
LOC105213937	chitinase-like protein Idgf4
LOC105216017	pupal cuticle protein C1B-like
LOC105216018	cuticle protein 12.5-like
LOC105216019	cuticle protein 16.5%2C isoform B-like
LOC105216022	cuticle protein 19.8-like
LOC105216023	cuticle protein 38-like
LOC105216028	cuticle protein 16.5%2C isoform A-like
LOC105216029	cuticle protein 16.5%2C isoform B-like
LOC105218487	chitinase-3-like protein 2
LOC105218843	pupal cuticle protein Edg-78E
