# Supplementary material for: A Chromosome-Scale Assembly of the Bactrocera cucurbitae Genome Provides Insight to the Genetic Basis of white pupae
Source: G3 (Bethesda). 2017 Apr 20;7(6):1927–40. doi: 10.1534/g3.117.040170 (PMC5473769; doi:10.1534/g3.117.040170)
Supplement: Supplementary file 13 [file 1927TableS4.pdf]

Table S4. Regression between position of gene orthologs in *D. melanogaster* and *B. cucurbitae*

**Muller element**	***B. cucurbitae* chr.**	***D. melanogaster* chr.**	R^2	**p-value**
A	3	X	0.04	$< 1e{-4}$
B	5	2L	0.08	$< 1e{-4}$
C	6	2R	0.01	$< 1e{-4}$
D	2	3L	0.23	$< 1e{-4}$
E	4	3R	0.05	$< 1e{-4}$
